# An Optimization-Based Meta-Learning Model for MRI Reconstruction with Diverse Dataset

**DOI:** 10.3390/jimaging7110231

**Published:** 2021-10-31

**Authors:** Wanyu Bian, Yunmei Chen, Xiaojing Ye, Qingchao Zhang

**Affiliations:** 1 Department of Mathematics, University of Florida, Gainesville, FL 32611, USA; yun@ufl.edu (Y.C.); qingchaozhang@ufl.edu (Q.Z.); 2 Department of Mathematics and Statistics, Georgia State University, Atlanta, GA 30303, USA; xye@gsu.edu

**Keywords:** MRI reconstruction, meta-learning, domain generalization

## Abstract

This work aims at developing a generalizable Magnetic Resonance Imaging (MRI) reconstruction method in the meta-learning framework. Specifically, we develop a deep reconstruction network induced by a learnable optimization algorithm (LOA) to solve the nonconvex nonsmooth variational model of MRI image reconstruction. In this model, the nonconvex nonsmooth regularization term is parameterized as a structured deep network where the network parameters can be learned from data. We partition these network parameters into two parts: a task-invariant part for the common feature encoder component of the regularization, and a task-specific part to account for the variations in the heterogeneous training and testing data. We train the regularization parameters in a bilevel optimization framework which significantly improves the robustness of the training process and the generalization ability of the network. We conduct a series of numerical experiments using heterogeneous MRI data sets with various undersampling patterns, ratios, and acquisition settings. The experimental results show that our network yields greatly improved reconstruction quality over existing methods and can generalize well to new reconstruction problems whose undersampling patterns/trajectories are not present during training.

## 1. Introduction

Deep learning methods have demonstrated promising performance in a variety of image reconstruction problems. However, deep learning models are often trained for specific tasks and require the training samples to follow the corresponding distribution. In particular, the source-domain/training samples and target-domain/testing samples need to be drawn from the same distribution [1,2,3,4]. In practice, these data sets are often collected at different sources and exhibit substantial heterogeneity, and thus the samples may follow related but different distributions in real-world applications [5,6]. Therefore, the robust and efficient training of deep neural networks using such data sets is theoretically important and practically relevant in the application of deep learning-based methods.

Meta-learning provides a unique paradigm to achieve robust and efficient neural network training [1,4,7,8,9,10]. Meta-learning is known as *learning-to-learn* and aims to quickly learn unseen tasks from the experience of learning episodes that cover the distribution of relevant tasks. In a multiple-task scenario, given a family of tasks, meta-learning has been proven to be a useful tool for extracting task-agnostic knowledge and improving the learning performance of new tasks from that family [11,12]. We leverage this feature of meta-learning for network training where the MRI training data are acquired by using different under-sampling patterns (e.g., Cartesian mask, Radial mask, Poisson mask), under-sampling ratios, and different settings of the scanning parameters, which result in different levels of contrast (e.g., T1-weighted, T2-weighted, proton-density (PD), and Flair). These data are vastly heterogeneous and can be considered as being from various tasks. Thus, our goal is to develop a robust and generalizable image reconstruction method in the meta-learning framework to leverage such large-scale heterogeneous data for MRI reconstruction.

Our approach can be outlined as follows. First, we introduce a variational model rendering a nonconvex nonsmooth optimization problem for image reconstruction. In our variational model, the regularization term is parameterized as a structured deep neural network where the network parameters can be learned during the training process. We then propose a learnable optimization algorithm (LOA) with rigorous convergence guarantees to solve this optimization problem. Then, we construct a deep reconstruction network by following this LOA exactly; namely, each phase of this LOA-induced network is exactly one iteration of the LOA. This approach is inspired by [13], but the LOA developed in the present work is computationally more efficient than that in [13]: the safeguard variable in this work is updated only if necessary, which can significantly reduce computational cost while retaining the convergence guarantee.

Second, to improve network robustness and mitigate the overfitting issue, we explicitly partition the network parameters of the regularization into two parts: a task-invariant part and a task-specific part. The former extracts common prior information of images from different tasks in the training data and learns the task-invariant component of the regularization. The latter, on the other hand, is obtained from another network which exploits proper task-specific components (also called meta-knowledge) of the regularization for different tasks. The hyperparameters of this network are also learned during training. Furthermore, we split the available data into two sets: the training set and the validation set. Then, we introduce a bilevel optimization model for learning network parameters. Specifically, the lower-level problem (also known as inner problem) finds the task-invariant part of the regularization with the fixed task-specific part on the training dataset, whereas the upper-level (outer) problem seeks for the optimal task-specific part of the regularization parameter using the validation dataset. This approach greatly increases the robustness of the learned regularization, meaning that the trained LOA-induced deep reconstruction network can generalize well to unseen tasks.

As demonstrated by our numerical experiments in Section 5, our proposed framework yields much improved image qualities using diverse data sets of various undersampling trajectories and ratios for MRI image reconstruction. The reason is that effective regularization can integrate common features and prior information from a variety of training samples from diverse data sets, but they need to be properly weighed against the data fidelity term obtained in specific tasks (i.e., undersampling trajectory and ratios). Our contributions can be summarized as follows:

1. An LOA inspired network architecture—our network architecture exactly follows a proposed LOA with guaranteed convergence. Thus, the network is more interpretable, parameter-efficient, and stable than existing unrolling networks.
2. Adaptive design of regularization—our adaptive regularizer consists of a task-invariant part and a task-specific part, both of which can be appropriately trained from data.
3. Improved network robustness and generalization ability—we improve the robustness of the network parameter training process by posing it as a bilevel optimization using training data in the lower-level and validation data in the upper-level. This approach also improves the generalization ability of the trained network so that it can be quickly adapted to image reconstruction with new unseen sampling trajectories and produces high-quality reconstructions.

The remainder of the paper is organized as follows. In Section 2, we discuss related work for both optimization-based meta-learning and deep unrolled networks for MRI reconstructions. We propose our meta-learning model and the neural network in Section 3 and describe the implementation details in Section 4. Section 5 provides the numerical results of the proposed method. Section 6 concludes the paper.

## 2. Related Work

In recent years, meta-learning methods have demonstrated promising results in various fields with different techniques [12]. Meta-learning techniques can be categorized into three groups [14,15,16]: metric-based methods [17,18,19], model-based methods [20,21,22,23], and optimization-based methods [8,24,25]. Optimization-based methods are often cast as a bilevel optimization problem and exhibit relatively better generalizability for wider task distributions. We mainly focus on optimization-based meta-learning in this paper. For more comprehensive literature reviews and developments of meta-learning, we refer the readers to the recent surveys [12,16].

Optimization-based meta-learning methods have been widely used in a variety of deep learning applications [8,24,25,26,27,28,29,30,31]. The network training problems in these meta-learning methods are often cast as the bilevel optimization of a leader variable and a follower variable. The constraint of the bilevel optimization is that the follower variable is optimal for the lower-level problem for each fixed leader variable, and the ultimate goal of bilevel optimization is to find the optimal leader variable (often, the corresponding optimal follower variable as well) that minimizes the upper-level objective function under the constraint. The lower-level problem is approximated by one or a few gradient descent steps in many existing optimization-based meta learning applications, such as Model-Agnostic Meta-Learning (MAML) [8], and a large number of followup works of MAML proposed to improve generalization using similar strategy [9,15,27,29,30,32,33,34]. Deep bilevel learning [35] seeks to obtain better generalization than when trained on one task and generalize well to another task. The model is used to optimize a regularized loss function to find network parameters from the training set and identify hyperparameters so that the network performs well on the validation dataset.

When the unseen tasks lie in inconsistent domains with the meta-training tasks, as revealed in [36], the generalization behavior of the meta-learner will be compromised. This phenomenon partially arises from the meta-overfitting on the already seen meta-training tasks, which is identified as a memorization problem in [34]. A meta-regularizer forked with information theory is proposed in [34] to handle the memorization problem by regulating the information dependency during the task adaption. MetaReg [4] decouples the entire network into the feature network and task network, where the meta-regularization term is only applied to the task network. They first update the parameters of the task network with a meta-train set to obtain the domain-aligned task network and then update the parameters of the meta-regularization term on the meta-test set to learn the cross-domain generalization. In contrast to MetaReg, Feature-Critic Networks [37] exploit the meta-regularization term to pursue a domain-invariant feature extraction network. The meta-regularization is designed as a feature-critic network that takes the extracted feature as an input. The parameters of the feature extraction network are updated by minimizing the new meta-regularized loss. The auxiliary parameters in the feature-critic network are learned by maximizing the performance gain over the non-meta case. To effectively evaluate the performance of the meta-learner, several new benchmarks [38,39,40] were developed under more realistic settings that operate well on diverse visual domains. As mentioned in [39], the generalization to unseen tasks within multimodal or heterogeneous datasets remains a challenge to the existing meta-learning methods.

The aforementioned methods pursue domain generalization for the classification networks that learned a regularization function to learn cross-domain generalization. Our proposed method was developed to solve the inverse problem, and we construct an adaptive regularization that not only learns the universal parameters among tasks but also the task-aware parameters. The designated adaptive regularizer assists the generalization ability of the deep model so that the well-trained model can perform well on heterogeneous datasets of both seen and unseen tasks.

## 3. Proposed Method

### 3.1. Preliminaries

We first provide the background of compressed sensing MRI (CS-MRI), the image reconstruction problem, and the learned optimization algorithm to solve the image reconstruction problem. CS-MRI accelerates MRI data acquisition by under-sampling the k-space (Fourier space) measurements. The under-sampled k-space measurement are related to the image by the following formula [41]:

(1) y=PFx+n,

where $y\in C^{p}$ represents the measurements in k-space with a total of *p* sampled data points, $x\in C^{N\times 1}$ is the MR image to be reconstructed with *N* pixels, $F\in C^{N\times N}$ is the 2D discrete Fourier transform (DFT) matrix, and $P\in R^{p\times N}$(p<N) is the binary matrix representing the sampling trajectory in k-space. n is the acquisition noise in k-space.

Solving x from (noisy) under-sampled data y according to (1) is an ill-posed problem. An effective strategy to elevate the ill-posedness issue is to incorporate prior information to the reconstruction. The variational method is one of the most effective ways to achieve this. The general framework of this method is to minimize an objective function that consists of a data fidelity term and a regularization term as follows:

(2) $$\bar{x}=\underset{x}{arg min}\frac{1}{2}{∥PFx-y∥}^{2}+R\left(x\right),$$

where the first term is data fidelity, which ensures consistency between the reconstructed x and the measured data y, and the second term R(x) is the regularization term, which introduces prior knowledge to the image to be reconstructed. In traditional variational methods, R(x) is a hand-crafted function such as Total Variation (TV) [42]. The advances of the optimization techniques allowed more effective algorithms to solve the variational models with theoretical justifications. However, hand-crafted regularizers may be too simple to capture subtle details and satisfy clinic diagnostic quality.

In recent years, we have witnessed the tremendous success of deep learning in solving a variety of inverse problems, but the interpretation and generalization of these deep-learning-based methods still remain the main concerns. As an improvement over generic black-box-type deep neural networks (DNNs), several classes of learnable optimization algorithms (LOAs) inspired neural networks, known as unrolling networks, which unfold iterative algorithms to multi-phase networks and have demonstrated promising solution accuracy and efficiency empirically [43,44,45,46,47,48,49,50]. However, many of them are only specious imitations of the iterative algorithms and hence lack the backbone of the variational model and any convergence guarantee.

In light of the substantial success of deep learning and the massive amount of training data now available, we can parameterize the regularization term as a deep convolutional neural network (CNN) that learns from training samples. LOA-induced reconstruction methods have been successfully applied to CS-MRI to solve inverse problems with a learnable regularizer:

(3) $$\underset{x}{arg min}\frac{1}{2}{∥PFx-y∥}^{2}+R\left(x;\Theta \right).$$

where R(x;Θ) is the regularization parameterized as a deep network with parameter Θ. Depending on the specific parametric form of R(x;Θ) and the optimization scheme used for unrolling, several unrolling networks have been proposed in recent years. For example, the variational network (VN) [51] was introduced to unroll the gradient descent algorithm and parametrize the regularization as a combination of linear filters and nonlinear CNNs. MoDL [52] proposed a weight sharing strategy in a recursive network to learn the regularization parameters by unrolling the conjugate gradient method. ADMM-Net [53] mimics the celebrated alternating direction method of multipliers; the regularizer is designed to be $L_{1}$-norm replaced by a piecewise linear function. ISTA-Net [54] considers the regularizer as the $L_{1}$-norm of a convolutional network. The network unrolls several phases iteratively, and each phase mimics one iteration of iterative shrinkage thresholding algorithm (ISTA) [55,56]. However, these networks only superficially mimic the corresponding optimization schemes, but they lack direct relations to the original optimization method or variational model and do not retain any convergence guarantee. In this work, we first develop a learnable optimization algorithm (LOA) for (3) with comprehensive convergence analysis and obtain an LOA-induced network by following the iterative scheme of the LOA exactly.

### 3.2. LOA-Induced Reconstruction Network

In this section, we first introduce a learned optimization algorithm (LOA) to solve (3) where the regularization network parameter Θ is fixed. As Θ is fixed in (3), we temporarily omit this in the derivation of the LOA below and write R(x;Θ) as R(x) for notation simplicity.

In this work, to incorporate sparsity along with the learned features, we parameterize the function R(x)=κ·r(x), where κ>0 is a weight parameter that needs be chosen properly depending on the specific task (e.g., noise level, undersampling ratio, etc.), and *r* is a regularizer parameterized as a composition of neural networks and can be adapted to a broad range of imaging applications. Specifically, we parameterize *r* as the composition of the $l_{2,1}$ norm and a learnable feature extraction operator g(x). That is, we set *r* in (11) to be

(4) $$r\left(x\right):={∥g\left(x\right)∥}_{2,1}=\sum_{j=1}^{m}∥g_{j}\left(x\right)∥.$$

Here, “:=” stands for “defined as”. $g\left(\cdot \right)=(g_{1}\left(\cdot \right),\cdots ,g_{m}\left(\cdot \right))$, $g_{j}\left(\cdot \right)=g_{j}\left(\cdot ;\theta \right)$ is parametrized as a convolutional neural network (CNN) for j=1,⋯,m, and θ is the learned and fixed network parameter in r(·;θ), as mentioned above. We also consider κ to be learned and fixed as θ for now, and we discuss how to learn both of them in the next subsection. We use a smooth activation function in g as formulated in (20), which renders g a smooth but nonconvex function. Due to the nonsmooth ${∥\cdot ∥}_{2,1}$, *r* is therefore a nonsmooth nonconvex function.

Since the minimization problem in (11) is nonconvex and nonsmooth, we need to derive an efficient LOA to solve it. Here, we first consider smoothing the $l_{2,1}$ norm that for any fixed g(x) is

(5) $$r_{\varepsilon }\left(x\right)=\sum_{j=1}^{m}\sqrt{∥g_{j}{\left(x\right)∥}^{2}+\varepsilon^{2}}-\varepsilon .$$

We denote $R_{\varepsilon }=\kappa \cdot r_{\varepsilon }$. The LOA derived here is inspired by the proximal gradient descent algorithm and iterates the following steps to solve the smoothed problem:

(6a) $$\begin{array}{cc} z_{t+1} & =x_{t}-\alpha_{t}\nabla f\left(x_{t}\right) \end{array}$$

(6b) $$\begin{array}{cc} x_{t+1} & ={\mathrm{prox}}_{\alpha_{t}R_{\varepsilon_{t}}}\left(z_{t+1}\right), \end{array}$$

where $\varepsilon_{t}$ denotes the smoothing parameter ε at the specific iteration *t*, and the proximal operator is defined as ${\mathrm{prox}}_{\alpha g}\left(b\right):={arg min}_{x}∥x-b∥+\alpha g\left(x\right)$ in (6b). A quick observation from (5) is that $R_{\varepsilon }\to R$ as ε diminishes, so later we intentionally push $\varepsilon_{t}\to 0$ at Line 16 in Algorithm 1. Then, one can readily show that $R_{\varepsilon }\left(x\right)\leq R\left(x\right)\leq R_{\varepsilon }\left(x\right)+\varepsilon$ for all *x* and ε>0. From Algorithm 1, line 16 automatically reduces ε, and the iterates will converge to the solution of the original nonsmooth nonconvex problem (11)—this is clarified precisely in the convergence analysis in Appendix A.

Since $R_{\varepsilon_{t}}$ is a complex function involving a deep neural network, its proximal operator does not have a closed form and cannot be computed easily in the subproblem in (6b). To overcome this difficulty, we consider to approximate $R_{\varepsilon_{t}}$ by

(7) $$\begin{array}{cc} {\hat{R}}_{\varepsilon_{t}}\left(z_{t+1}\right) & =R_{\varepsilon_{t}}\left(z_{t+1}\right)+\langle \nabla R_{\varepsilon_{t}}\left(z_{t+1}\right),x-z_{t+1}\rangle +\frac{1}{2\beta_{t}}{∥x-z_{t+1}∥}^{2}. \end{array}$$

Then, we update $u_{t+1}={\mathrm{prox}}_{\alpha_{t}{\hat{R}}_{\varepsilon_{t}}}\left(z_{t+1}\right)$ to replace (6b); therefore, we obtain

(8) $$u_{t+1}=z_{t+1}-\tau_{t}\nabla R_{\varepsilon_{t}}\left(z_{t+1}\right),\;\mathrm{where}\;\tau_{t}=\frac{\alpha_{t}\beta_{t}}{\alpha_{t}+\beta_{t}}.$$

In order to guarantee the convergence of the algorithm, we introduce the standard gradient descent of $\phi_{\varepsilon_{t}}$ (where $\phi_{\varepsilon_{t}}:=f+R_{\varepsilon_{t}}$) at x:

(9) $$v_{t+1}=\underset{x}{arg min}\langle \nabla f\left(x_{t}\right),x-x_{t}\rangle +\langle \nabla R_{\varepsilon }\left(x_{t}\right),x-x_{t}\rangle +\frac{1}{2\alpha_{t}}{∥x-x_{t}∥}^{2},$$

which yields

(10) $$v_{t+1}=x_{t}-\alpha_{t}\nabla \phi_{\varepsilon_{t}}\left(x_{t}\right),$$

to serve as a safeguard for the convergence. Specifically, we set $x_{t+1}=u_{t+1}$ if $\phi_{\varepsilon_{t}}\left(u_{t+1}\right)\leq \phi_{\varepsilon_{t}}\left(v_{t+1}\right)$; otherwise, we set $x_{t+1}=v_{t+1}$. Then, we repeat this process.

Our algorithm is summarized in Algorithm 1. The prior term with unknown parameters has the exact residual update itself which improves the learning and training process [57]. The condition checking in Line 5 is introduced to make sure that it is in the energy descending direction. Once the condition in Line 5 fails, the process moves to $v_{t+1}$, and the line search in Line 12 guarantees that the appropriate step size can be achieved within finite steps to make the function value decrease. From Line 3 to Line 14, we consider that it solves a problem of minimizing $\phi_{\varepsilon_{t}}$ with $\varepsilon_{t}$ fixed. Line 15 is used to update the value of $\varepsilon_{t}$ depending on a reduction criterion. The detailed analysis of this mechanism and in-depth convergence justification is shown in Appendix A. The corresponding unrolling network exactly follows Algorithm 1 and thus shares the same convergence property. Compared to LDA [13], which computes both candidates $u_{t+1}$, $v_{t+1}$ at every iteration and then chooses the one that achieves a smaller function value, we propose the criteria above in Line 5 for updating $x_{t+1}$, which potentially saves extra computational time for calculating the candidate $v_{t+1}$ and potentially mitigates the frequent alternations between the two candidates. Besides, the smoothing method proposed in this work is more straightforward than smoothing in dual space [13] while still keeping provable convergence, as shown in Theorem A5.

The proposed LOA-induced network is a multi-phase network whose architecture exactly follows the proposed LOA (Algoirthm 1) in the way that each phase corresponds to one iteration of the algorithm. Specifically, we construct a deep network, denoted by $F_{\Theta }$, that follows Algorithm 1 exactly for a user-specified number of *T* iterations. Here, Θ denotes the set of learnable parameters in $F_{\Theta }$, which includes the regularization network parameter θ, weight κ, and other algorithmic parameters of Algorithm 1. Therefore, for any input under-sampled k-space measurement y, $F_{\Theta }\left(y\right)$ executes the LOA (Algorithm 1) for *T* iterations and generates an approximate solution of the minimization problem (11):

(11) $$F_{\Theta }\left(y\right)\approx \underset{x}{arg min}\left\{\phi_{\Theta }\left(x,y\right):=f\left(x,y\right)+R\left(x;\Theta \right)\right\}.$$

where we use “≈” since $F_{\Theta }$ follows only finitely many steps of the optimization algorithm to approximate the solution. It is worth emphasizing that this approach can be readily applied to a much broader class of image reconstruction problems as long as *f* is (possibly nonconvex and) continuously differentiable with the Lipschitz continuous gradient. In the next subsection, we develop a meta-learning based approach for the robust training of the network parameter Θ.

### 3.3. Bilevel Optimization Algorithm for Network Training

In this section, we consider the parameter training problem of the LOA-induced network $F_{\Theta }$. Specifically, we develop a bilevel optimization algorithm to train our network parameters Θ from diverse data sets to improve network robustness and generalization ability.

**Algorithm 1:** Algorithmic Unrolling Method with Provable Convergence
1:**Input:** Initial $x_{0}$, 0<ρ, γ<1, and $\varepsilon_{0}$, σ>0. Max total phases *T* or tolerance $\epsilon_{\mathrm{tol}}>0$. 2:
**for** t=0,1,2,⋯,T−1 **do**
3: $z_{t+1}=x_{t}-\alpha_{t}\nabla f\left(x_{t}\right)$4: $u_{t+1}=z_{t+1}-\tau_{t}\nabla R_{\varepsilon_{t}}\left(z_{t+1}\right)$, 5: **if** $∥\nabla \phi_{\varepsilon_{t}}\left(x_{t}\right)∥\leq a∥u_{t+1}-x_{t}∥\;\mathrm{and}\;\phi_{\varepsilon_{t}}\left(u_{t+1}\right)-\phi_{\varepsilon_{t}}\left(x_{t}\right)\leq -\frac{1}{a}{∥u_{t+1}-x_{t}∥}^{2}$ **then**6:  set $x_{t+1}=u_{t+1}$, 7: **else**8:  $v_{t+1}=x_{t}-\alpha_{t}\nabla \phi_{\varepsilon_{t}}\left(x_{t}\right)$, 9:  **if** $\phi_{\varepsilon_{t}}\left(v_{t+1}\right)-\phi_{\varepsilon_{t}}\left(x_{t}\right)\leq -\frac{1}{a}{∥v_{t+1}-x_{t}∥}^{2}$ holds **then**10:   set $x_{t+1}=v_{t+1}$, 11:  **else**12:   update $\alpha_{t}\leftarrow \rho \alpha_{t}$, then **go to** 8, 13:  **end if**14: **end if**15: **if**  $∥\nabla \phi_{\varepsilon_{t}}\left(x_{t+1}\right)∥<\sigma \gamma \varepsilon_{t}$, set $\varepsilon_{t+1}=\gamma \varepsilon_{t}$; **otherwise**, set $\varepsilon_{t+1}=\varepsilon_{t}$.16: **if** $\sigma \varepsilon_{t}<\epsilon_{\mathrm{tol}}$, terminate.17:
**end for**
18:**Output:** $x_{t}$.


Recall that the LOA-induced network $F_{\Theta }$ exactly follows Algorithm 1, which is designed to solve the variational model (11) containing learnable regularization R(x;Θ). As shown in Section 3.2, we design R(x;Θ)=κ·r(x;Θ), where *r* is learned to capture the intrinsic property of the underlying common features across all different tasks. To account for the large variations in the diverse training/validation data sets, we introduce a task-specific parameter $\omega_{i}$ to approximate the proper κ for the *i*th task. Specifically, for the *i*th task, the weight κ is set to $\sigma \left(\omega_{i}\right)\in \left(0,1\right)$, where σ(·) is the sigmoid function. Therefore, $\kappa =\sigma (\omega_{i})$ finds the proper weight of *r* for the *i*-th task according to its specific sampling ratio or pattern. The parameters $\omega_{i}$ are to be optimized in conjunction with Θ through the hyperparameter tuning process below.

Suppose that we are given M data pairs ${\left\{\left(y_{m},x_{m}^{*}\right)\right\}}_{m=1}^{M}$ for the use of training and validation, where $y_{m}$ is the observation, which is the partial k-space data in our setting, and $x_{m}^{*}$ is the corresponding ground truth image. The data pairs are then sampled into N tasks ${\left\{D_{\tau_{i}}\right\}}_{i=1}^{N}$, where each $D_{\tau_{i}}$ represents the collection of data pairs in the specific task $\tau_{i}$. In each task $\tau_{i}$, we further divide the data into the task-specific training set $D_{\tau_{i}}^{tr}$ and validation set $D_{\tau_{i}}^{val}$. The architecture of our base network exactly follows the LOA (Algorithm 1) developed in the previous section with learnable parameters θ and a task-specific parameter $\omega_{i}$ for the *i*th task. More precisely, for one data sample denoted by $\left(y_{j}^{\left(i\right)},x{*}_{j}^{\left(i\right)}\right)$ in task $\tau_{i}$ with index *j*, we propose the algorithmic unrolling network for task $\tau_{i}$ as

(12) $$F_{\theta ,\omega_{i}}\left(y_{j}^{\left(i\right)}\right)\approx \underset{x}{arg min}f\left(x,y_{j}^{\left(i\right)}\right)+\sigma \left(\omega_{i}\right)r\left(x;\theta \right),$$

where θ denotes the learnable common parameters across different tasks with task-invariant representation, whereas $\omega_{i}$ is a task-specific parameter for task $\tau_{i}$. The weight $\sigma (\omega_{i})$ represents the weight of *r* associated with the specific task $\tau_{i}$. In our proposed network, Θ is the collection of $\left(\theta ,\omega_{i}\right)$ for task i=1,⋯N. We denote ω to be the set ${\left\{\omega_{i}\right\}}_{i=1}^{N}$. The detailed architecture of this network is illustrated in Section 3.2. We define the task-specific loss

(13) $$\ell_{\tau_{i}}\left(\theta ,\omega_{i};D_{\tau_{i}}\right):=\sum_{j=1}^{|D_{\tau_{i}}|}\ell \left(F_{\theta ,\omega_{i}}\left(y_{j}^{\left(i\right)}\right),x{*}_{j}^{\left(i\right)}\right),$$

where $|D_{\tau_{i}}|$ represents the cardinality of $D_{\tau_{i}}$ and

(14) $$\ell \left(F_{\theta ,\omega_{i}}\left(y_{j}^{\left(i\right)}\right),x{*}_{j}^{\left(i\right)}\right):=\frac{1}{2}{∥F_{\theta ,\omega_{i}}\left(y_{j}^{\left(i\right)}\right)-x{*}_{j}^{\left(i\right)}∥}^{2}.$$

For the sake of preventing the proposed model from overfitting the training data, we introduce a novel learning framework by formulating the network training as a bilevel optimization problem to learn ω and θ in (12) as

(15a) $$\begin{array}{cc} \min_{\theta ,\;\omega =\{\omega_{i}:i\in \left[N\right]\}}\; & \sum_{i=1}^{N}\ell_{\tau_{i}}\left(\theta \left(\omega \right),\omega_{i};D_{\tau_{i}}^{val}\right) \end{array}$$

(15b) $$\begin{array}{cc} s.t.\;\; & \theta \left(\omega \right)=\underset{\theta }{arg min}\sum_{i=1}^{N}\ell_{\tau_{i}}\left(\theta ,\omega_{i};D_{\tau_{i}}^{tr}\right). \end{array}$$

In (15), the lower-level optimization learns the task-invariant parameters θ of the feature encoder with the fixed task-specific parameter $\omega_{i}$ on the training dataset, and the upper-level adjusts the task-specific parameters $\left\{\omega_{i}\right\}$ so that the task-invariant parameters θ can perform robustly on the validation dataset as well. For simplicity, we omit the summation and redefine $L\left(\theta ,\omega ;D\right):=\sum_{i=1}^{N}\ell_{\tau_{i}}\left(\theta ,\omega ;D\right)$ and then briefly rewrite (15) as

(16) $$\min_{\theta ,\omega }L\left(\theta \left(\omega \right),\omega ;D^{val}\right)\;\;\;\;\;s.t.\;\;\theta \left(\omega \right)=\underset{\theta }{arg min}L\left(\theta ,\omega ;D^{tr}\right).$$

Then, we relax (16) into a single-level constrained optimization where the lower-level problem is replaced with its first-order necessary condition following [58]

(17) $$\min_{\theta ,\omega }L\left(\theta ,\omega ;D^{val}\right)\;\;\;\;\;s.t.\;\;\nabla_{\theta }L\left(\theta ,\omega ;D^{tr}\right)=0.$$

which can be further approximated by an unconstrained problem by a penalty term as

(18) $$\min_{\theta ,\omega }\{\tilde{L}\left(\theta ,\omega ;D^{tr},D^{val}\right):=L\left(\theta ,\omega ;D^{val}\right)+\frac{\lambda }{2}{∥\nabla_{\theta }L\left(\theta ,\omega ;D^{tr}\right)∥}^{2}\}.$$

We adopt the stochastic gradients of the loss functions on mini-batch data sets in each iteration. In our model, we need to include the data pairs of multiple tasks in one batch; therefore, we propose the cross-task mini-batches when training. At each training iteration, we randomly sample the training data pairs $B_{\tau_{i}}^{tr}={\left\{\left(y_{j}^{\left(i\right)},x{*}_{j}^{\left(i\right)}\right)\in D_{\tau_{i}}^{tr}\right\}}_{j=1}^{J^{tr}}$ and the validation pairs $B_{\tau_{i}}^{val}={\left\{\left(y_{j}^{\left(i\right)},x{*}_{j}^{\left(i\right)}\right)\in D_{\tau_{i}}^{val}\right\}}_{j=1}^{J^{val}}$ on each task $\tau_{i}$. Then, the overall training and validation mini-batches $B^{tr}$ and $B^{val}$ used in every training iteration are composed of the sampled data pairs from the entire set of tasks; i.e., $B^{tr}={⋃}_{i=1}^{N}\left\{B_{\tau_{i}}^{tr}\right\}$ and $B^{val}={⋃}_{i=1}^{N}\left\{B_{\tau_{i}}^{val}\right\}$. Thus in each iteration, we have $N\cdot J^{tr}$ and $N\cdot J^{val}$ data pairs used for training and validation, respectively. To solve the minimization problem (17), we utilize the stochastic mini-batch alternating direction method summarized in Algorithm 2, which is modified from [58].

As analyzed in [58], this penalty-type method has linear time complexity without computing the Hessian of the low level and only requires a constant space since we only need to store the intermediate θ,ω at each training iteration, which is suitable for solving the large-scale bilevel optimization problem. Algorithm 2 relaxes the bi-level optimization problem to a single-level constrained optimization problem by using the first-order necessary condition, which is not equivalent to the original problem but is much easier and efficient to solve. In the inner-loop (Line 5–9) of Algorithm 2, we continue minimizing the converted single-level optimization function (18) with respect to θ for *K* steps and then ω once alternatively until the condition with tolerance δ in Line 5 fails. The basic idea behind the condition in Line 5 arises from the first-order necessary condition as we would like to push the gradient of $\tilde{L}$ toward 0. Furthermore, at Line 11 of the outer loop (Line 2–11), we decrease the tolerance δ. Combining Line 5 and 11 guarantees that each time the inner loop terminates, the gradients of $\tilde{L}$ with respect to θ and ω become increasingly close to 0. The parameter $\delta_{tol}$ is used to control the accuracy of the entire algorithm, and the outer-loop will terminate when δ is sufficiently small (i.e., $\delta \leq \delta_{tol}$). In addition, λ is the weight for the second constraint term of (18); in the beginning, we set λ to be small to achieve a quick starting convergence, then gradually increase its value to emphasize the constraint.
**Algorithm 2:** Stochastic mini-batch alternating direction penalty method to solve problem (17)1:**Input**$D_{\tau_{i}}^{tr}$, $D_{\tau_{i}}^{val}$, $\delta_{tol}>0$.2:**Initialize**θ, ω, δ, λ>0 and $\nu_{\delta }\in \left(0,1\right)$, $\nu_{\lambda }>1$.3:**while**$\delta >\delta_{tol}$**do**4:    Sample cross-task training batch $B^{tr}={⋃}_{i=1}^{N}{\left\{\left(y_{j}^{\left(i\right)},x{*}_{j}^{\left(i\right)}\right)\in D_{\tau_{i}}^{tr}\right\}}_{j=1:J^{tr}}$5:    Sample cross-task validation batch $B^{val}={⋃}_{i=1}^{N}{\left\{\left(y_{j}^{\left(i\right)},x{*}_{j}^{\left(i\right)}\right)\in D_{\tau_{i}}^{val}\right\}}_{j=1:J^{val}}$6:    **while** $∥\nabla_{\theta }\tilde{L}\left(\theta ,\omega ;B^{tr},B^{val}\right){∥}^{2}+{∥\nabla_{\omega }\tilde{L}\left(\theta ,\omega ;B^{tr},B^{val}\right)∥}^{2}>\delta$ **do**7:        **for** k=1,2,⋯,K (inner loop) **do**8:           $\theta \leftarrow \theta -\rho_{\theta }^{k}\nabla_{\theta }\tilde{L}\left(\theta ,\omega ;B^{tr},B^{val}\right)$9:        **end for**10:        $\omega \leftarrow \omega -\rho_{\omega }\nabla_{\omega }\tilde{L}\left(\theta ,\omega ;B^{tr},B^{val}\right)$11:    **end while**12:    **update** $\delta \leftarrow \nu_{\delta }\delta$, $\;\lambda \leftarrow \nu_{\lambda }\lambda$13:**end while**14:**output:**θ,ω.

## 4. Implementation

### 4.1. Feature Extraction Operator

We set the feature extraction operator g to be a vanilla *l*-layer CNN with the component-wise nonlinear activation function φ and no bias, as follows:

(19) $$g\left(x\right)=w_{l}*\varphi \cdots \;\varphi \left(w_{3}*\varphi \left(w_{2}*\varphi \left(w_{1}*x\right)\right)\right),$$

where ${\left\{w_{q}\right\}}_{q=1}^{l}$ denote the convolution weights consisting of *d* kernels with identical spatial kernel size, and ∗ denotes the convolution operation. Here, φ is constructed to be the smoothed rectified linear unit as defined below:

(20) $$\varphi \left(x\right)=\left\{\begin{array}{cc} 0, & if\;x\leq -\delta ,\\ \frac{1}{4\delta }x^{2}+\frac{1}{2}x+\frac{\delta }{4}, & if\;-\delta <x<\delta ,\\ x, & if\;x\geq \delta , \end{array}\right.$$

where the prefixed parameter δ is set to be 0.001 in our experiment. The default configuration of the feature extraction operator is set as follows: the feature extraction operator g consists of l=3 convolution layers and all convolutions are with 4 kernels of a spatial size of 3×3.

### 4.2. Setups

As our method introduces an algorithmic unrolling network, there exists a one-to-one correspondence between the algorithm iterations and the neural network phases (or blocks). Each phase of the forward propagation can be viewed as one algorithm iteration, which motivates us to imitate the iterating of the optimization algorithm and use a stair training strategy [13]. At the first stage, we start training the network parameters using one phase, then after the the loss converges, we add more phases (one phase each time) then continue the training process. We repeat this procedure and stop it when the loss does not decrease any further when we add more blocks. We minimize the loss for 100 epochs/iterations each time using the SGD-based optimizer Adam [59] with $\beta_{1}=0.9$, $\beta_{2}=0.999$, and the initial learning rate set to $10^{-3}$ as well as a mini-batch size of 8. The Xavier Initializer [60] is used to initialize the weights of all convolutions. The initial smoothing parameter $\varepsilon_{0}$ is set to be 0.001 and then learned together with other network parameters. The input $x_{0}$ of the unrolling network is obtained by the zero-filling strategy [61]. The deep unrolling network was implemented using the Tensorflow toolbox [62] in the Python programming language.

## 5. Numerical Experiments

### 5.1. Dataset

To validate the performance of the proposed method, the data we used were from Multimodal Brain Tumor Segmentation Challenge 2018 [63], in which the training dataset contains four modalities (T1, T1${}_{c}$, T2 and FLAIR )scanned from 285 patients and the validation dataset contains images from 66 patients, each with a volume size of 240×240×155. Each modality consists of two types of gliomas: 75 volumes of low-grade gliomas (LGG) and 210 volumes of high-grade gliomas (HGG). Our implementation involved HGG MRI in two modalities—T1 and T2 images—and we chose 30 patients from each modality in the training dataset to train our network. In the validation dataset, we randomly picked 15 patients as our validation data and 6 patients in the training dataset as testing data, which were distinct from our training set and validation set. We cropped the 2D image size to be 160×180 in the center region and picked 10 adjacent slices in the center of each volume, resulting in a total of 300 images as our training data, 150 images as our validation data, and a total of 60 images as testing data. The amount of data mentioned here is for a single task, but since we emploedy multi-task training, the total number of images in each dataset should be multiplied by the number of tasks. For each 2D slice, we normalized the spatial intensity by dividing the maximum pixel value.

### 5.2. Experiment Settings

**Figure 1:**
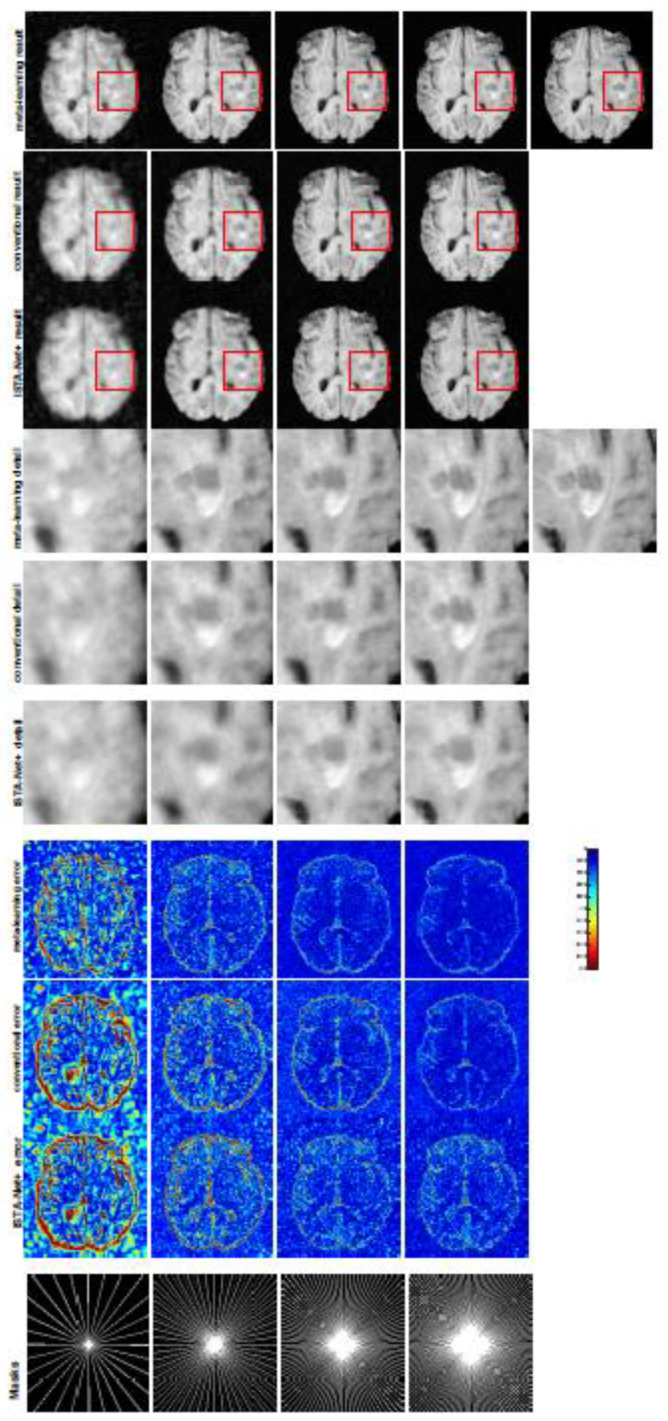
The pictures (from top to bottom) display the reconstruction results, zoomed-in details, point-wise errors with a color bar, and associated **radial** masks for meta-learning, conventional learning, and ISTA-Net${}^{+}$ with four different CS ratios of 10%, 20%, 30%, 40% (from left to right). The top-right image is the ground truth fully-sampled image.

**Figure 2:**
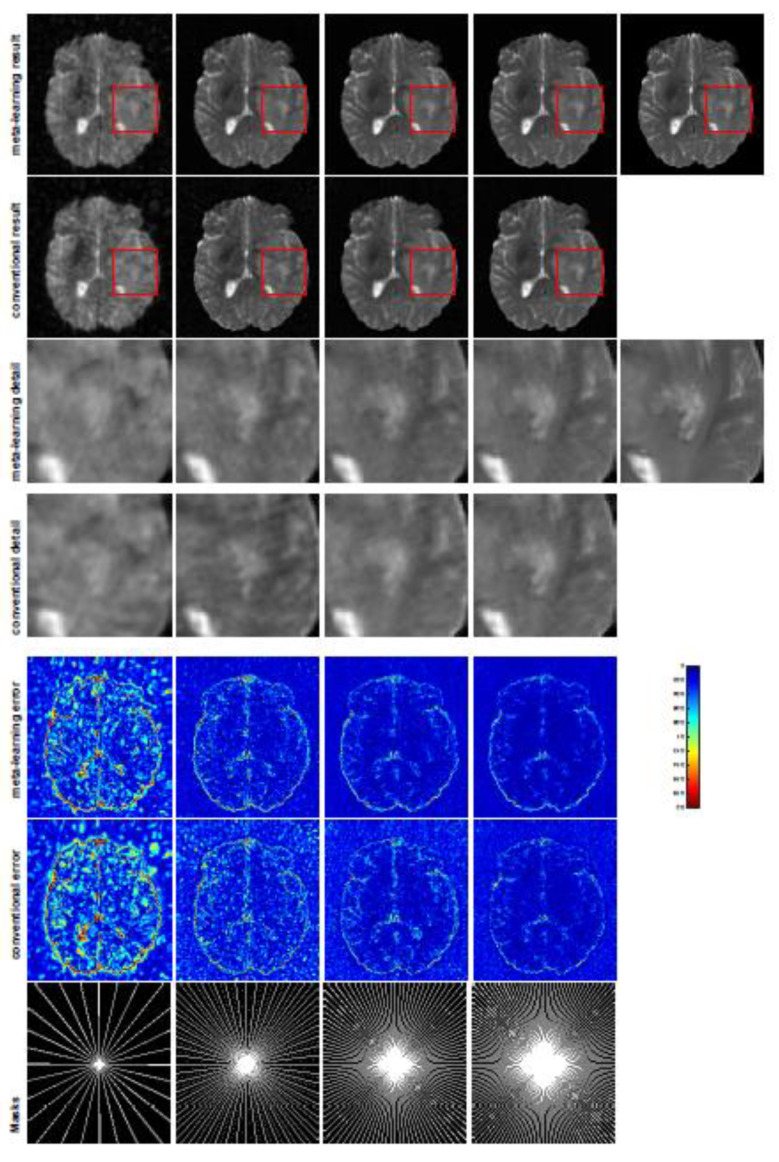
The pictures (from top to bottom) display the T2 brain image reconstruction results, zoomed-in details, point-wise errors with a color bar, and associated **radial** masks for meta-learning and conventional learningwith four different CS ratios of 10%, 20%, 30%, 40% (from left to right). The top-right iage is the ground truth fully-sampled image.

**Figure 3:**
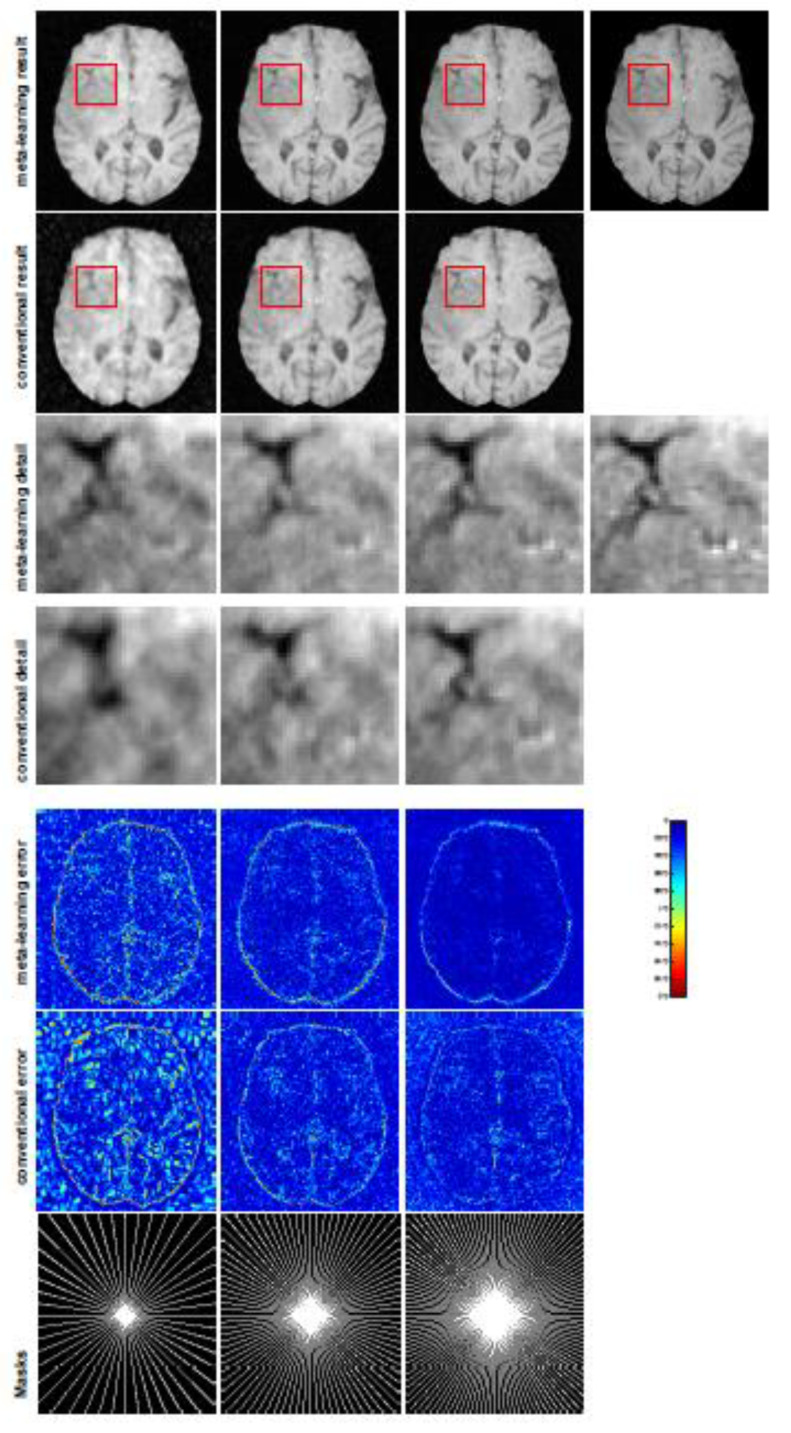
The pictures (from top to bottom) display the T1 brain image reconstruction results, zoomed-in details, point-wise errors with a color bar, and associated **radial** masks for meta-learning and conventional learning. Meta-learning was trained with CS ratios of 10%, 20%, 30%, and 40% and tested with three different unseen CS ratios of 15%, 25%, and 35% (from left to right). Conventional learning was trained and tested with the same CS ratios of 15%, 25%, and 35%. The top-right image is the ground truth fully-sampled image.

**Figure 4:**
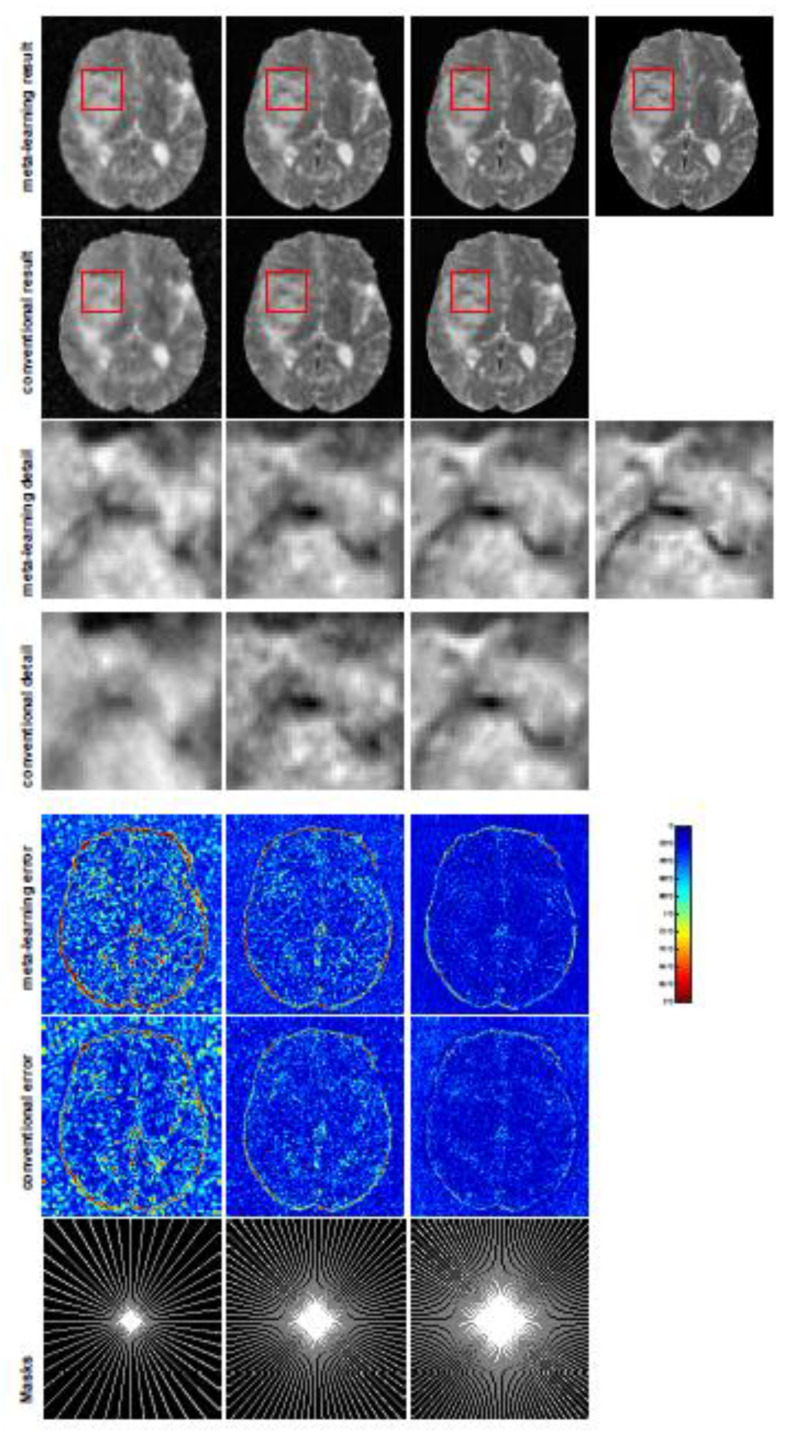
The pictures (from top to bottom) display the T2 brain image reconstruction results, zoomed-in details, point-wise errors with a color bar, and associated **radial** masks for meta-learning and conventional learning. Meta-learning was trained with CS ratios of 10%, 20%, 30%, and 40% and test edwith three different unseen CS ratios of 15%, 25%, and 35% (from left to right). Conventional learning was trained and tested with the same CS ratios of 15%, 25%, and 35%. The top-right image is the ground truth fully-sampled image.

**Figure 5:**
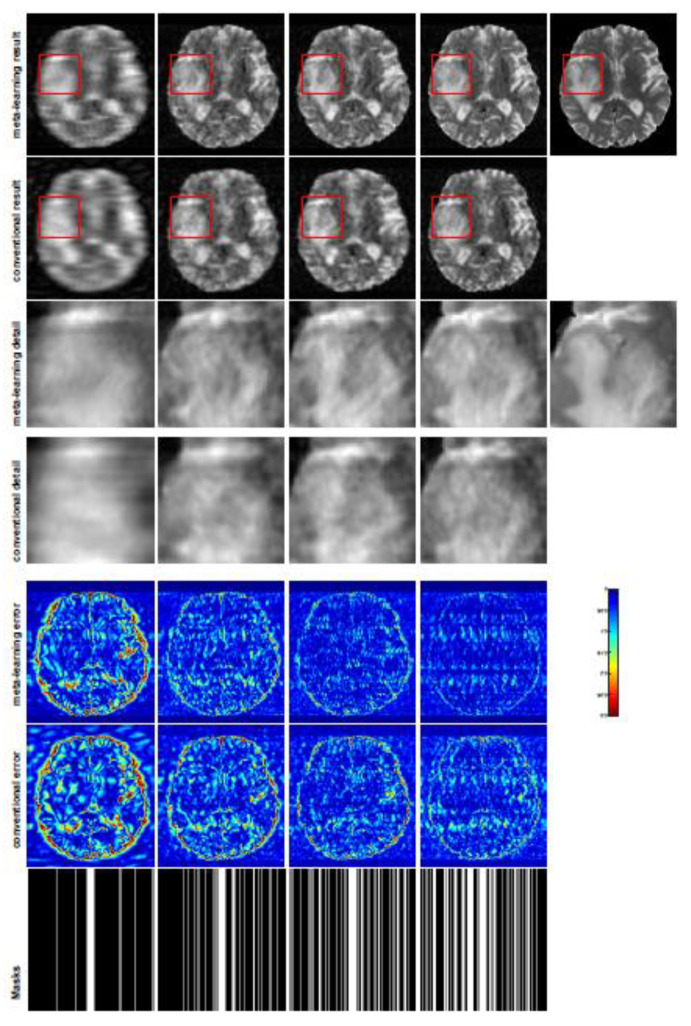
The pictures (from top to bottom) display the T2 brain image reconstruction results, zoomed-in details, point-wise errors with a color bar, and associated **Cartesian** masks for meta-learning and conventional learningwith four different CS ratios of 10%, 20%, 30%, and 40% (from left to right). The top-right image is the ground truth fully-sampled image.

All the experiments were implemented on a Windows workstation with an Intel Core i9 CPU at 3.3GHz and an Nvidia GTX-1080Ti GPU with 11 GB of graphics card memory via TensorFlow [64]. The parameters in the proposed network were initialized by using Xavier initialization [65]. We trained the meta-learning network with four tasks synergistically associated with four different CS ratios—10%, 20%, 30%, and 40%—and tested the well-trained model on the testing dataset with the same masks of these four ratios. We used 300 training data for each CS ratio, amounting to a total of 1200 images in the training dataset. The results for T1 and T2 MR reconstructions are shown in Table 1 and Table 2, respectively. The associated reconstructed images are displayed in Figure 1 and Figure 2. We also tested the well-trained meta-learning model on unseen tasks with radial masks for unseen ratios of 15%, 25%, and 35% and random Cartesian masks with ratios of 10%, 20%, 30%, and 40%. The task-specific parameters for the unseen tasks were retrained for different masks with different sampling ratios individually with fixed task-invariant parameters θ. In this experiments, we only needed to learn $\omega_{i}$ for three unseen CS ratios with radial mask and four regular CS ratios with Cartesian masks. The experimental training proceeded with fewer data and iterations, where we used 100 MR images with 50 epochs. For example, to reconstruct MR images with a CS ratio of 15% from the radial mask, we fixed the parameter θ and retrained the task-specific parameter ω on 100 raw data points with 50 epochs, then tested with renewed ω on our testing data set with raw measurements sampled from the radial mask with a CS radial of 15%. The results associated with radial masks are shown in Table 3 and Table 4, Figure 3 and Figure 4 for T1 and T2 images, respectively. The results associated with Cartesian masks are listed in Table 5 and reconstructed images are displayed in Figure 5.

We compared our proposed meta-learning method with conventional supervised learning, which was trained with one task at each time and only learned the task-invariant parameter θ without the task-specific parameter $\omega_{i}$. The forward network of conventional learning unrolled Algorithm 1 with 11 phases, which was the same as meta-learning. We merged the training set and validation set, resulting in 450 images for the training of the conventional supervised learning. The training batch size was set as 25 and we applied a total of 2000 epochs, while in meta-learning, we applied 100 epochs with a batch size of 8. The same testing set was used in both meta-learning and conventional learning to evaluate the performance of these two methods.

We made comparisons between meta-learning and the conventional network on the seven different CS ratios (10%, 20%, 30%, 40%, 15%, 25%, and 35%) in terms of two types of random under-sampling patterns: radial sampling mask and Cartesian sampling mask. The parameters for both meta-learning and conventional learning networks were trained via the Adam optimizer [59], and they both learned the forward unrolled task-invariant parameter θ. The network training of the conventional method used the same network configuration as the meta-learning network in terms of the number of convolutions, depth and size of CNN kernels, phase numbers and parameter initializer, etc. The major difference in the training process between these two methods is that meta-learning is performed for multi-tasks by leveraging the task-specific parameter $\omega_{i}$ learned from Algorithm 2, and the common features among tasks are learned from the feed-forward network that unrolls Algorithm 1, while conventional learning solves the task-specific problem by simply unrolling the forward network via Algorithm 1, where both training and testing are implemented on the same task. To investigate the generalizability of meta-learning, we tested the well-trained meta-learning model on MR images in different distributions in terms of two types of sampling masks with various trajectories. The training and testing of conventional learning were applied with the same CS ratios; that is, if the conventional method was trained with a CS ratio 10%, then it was also tested on a dataset with a CS ratio of 10%, etc.

Because MR images are represented as complex values, we applied complex convolutions [66] for each CNN; that is, every kernel consisted of a real part and imaginary part. Three convolutions were used in g, where each convolution contained four filters with a spatial kernel size of 3×3. In Algorithm 1, a total of 11 phases can be achieved if we set the termination condition $\epsilon_{\mathrm{tol}}=1\times 10^{-3}$, and the parameters of each phase are shared except for the step sizes. For the hyperparameters in Algorithm 1, we chose an initial learnable step size $\alpha_{0}=0.01,\tau_{0}=0.01,\varepsilon_{0}=0.001$, and we set prefixed values of $a=10^{5},\sigma =10^{3},\rho =0.9$, and γ=0.9. The principle behind the choices of those parameters is based on the convergence of the algorithm and effectiveness of the computation. The parameter 0<ρ<1 is the reduction rate of the step size during the line search used to guarantee the convergence. The parameter 0<γ<1 at step 15 is the reduction rate for ε. In Algorithm 1, from step 2 to step 14, the smoothing level ε is fixed. When the gradient of the smoothed function is small enough, we reduce ε by a fraction factor γ to find an approximate accumulation point of the original nonsmooth nonconvex problem. We chose a larger *a* in order to have more iterations *k* for which $u_{k+1}$ satisfies the conditions in step 5, so that there would be fewer iterations requiring the computation of $v_{k+1}$. Moreover, the scheme for computing $u_{k+1}$ is in accordance with the residual learning architecture that has been proven effective for reducing training error.

In Algorithm 2, we set $\nu_{\delta }=0.95$ and the parameter δ was initialized as $\delta_{0}=1\times 10^{-3}$ and stopped at value $\delta_{tol}=4.35\times 10^{-6}$, and a total of 100 epochs were performed. To train the conventional method, we set 2000 epochs with the same number of phases, convolutions, and kernel sizes as used to train the meta-learning approach. The initial λ was set as $1\times 10^{-5}$ and $\nu_{\lambda }=1.001$.

We evaluated our reconstruction results on the testing data sets using three metrics: peak signal-to-noise ratio (PSNR) [67], structural similarity (SSIM) [68], and normalized mean squared error (NMSE) [69]. The following formulations compute the PSNR, SSIM, and NMSE between the reconstructed image x and ground truth $x^{*}$. PSNR can be induced by the mean square error (MSE) where

(21) $$PSNR\left(x,x^{*}\right)=20\log_{10}\left(\frac{\max(|x^{*}|)}{\sqrt{MSE(x,x^{*})}}\right),$$

where *N* is the total number of pixels of the ground truth and MSE is defined by $MSE\left(x,x^{*}\right)=\frac{1}{N}{∥x^{*}-x∥}^{2}$.

(22) $$SSIM\left(x,x^{*}\right)=\frac{\left(2\mu_{x}\mu_{x^{*}}+C_{1}\right)\left(2\sigma_{xx^{*}}+C_{2}\right)}{\left(\mu_{x}^{2}+\mu_{x^{*}}^{2}+C_{1}\right)\left(\sigma_{x}^{2}+\sigma_{x^{*}}^{2}+C_{2}\right)},$$

where $\mu_{x},\mu_{x^{*}}$ represent local means, $\sigma_{x},\sigma_{x^{*}}$ denote standard deviations, $\sigma_{xx^{*}}$ represents the covariance between x and $x^{*}$, $C_{1}={\left(k_{1}L\right)}^{2},C_{2}={\left(k_{2}L\right)}^{2}$ are two constants which avoid the zero denominator, and $k_{1}=0.01,k_{2}=0.03$. *L* is the largest pixel value of MR image.

(23) $$NMSE\left(x,x^{*}\right)=\frac{∥x-x^{*}{∥}_{2}^{2}}{{∥x∥}_{2}^{2}},$$

where NMSE is used to measure the mean relative error. For detailed information of these three metrics mentioned above, please refer to [67,68,69].

### 5.3. Experimental Results with Different CS Ratios in Radial Mask

In this section, we evaluate the performance of well-trained meta-learning and conventional learning approaches. Table 1, Table 2 and Table 5 report the quantitative results of averaged numerical performance with standard deviations and associated descaled task-specific meta-knowledge $\sigma (\omega_{i})$. From the experiments implemented with radial masks, we observe that the average PSNR value of meta-learning improved by 1.54 dB in the T1 brain image for all four CS ratios compared with the conventional method, and for the T2 brain image, the average PSNR of meta-learning improved by 1.46 dB. Since the general setting of meta-learning aims to take advantage of the information provided from each individual task, with each task associated with an individual sampling mask that may have complemented sampled points, the performance of the reconstruction from each task benefits from other tasks. Smaller CS ratios will inhibit the reconstruction accuracy, due to the sparse undersampled trajectory in raw measurement, while meta-learning exhibits a favorable potential ability to solve this issue even in the situation of insufficient amounts of training data.

In general supervised learning, training data need to be in the same or a similar distribution; heterogeneous data exhibit different structural variations of features, which hinder CNNs from extracting features efficiently. In our experiments, raw measurements sampled from different ratios of compressed sensing display different levels of incompleteness; these undersampled measurements do not fall in the same distribution but they are related. Different sampling masks are shown at the bottom of Figure 1 and Figure 3, and these may have complemented sampled points, in the sense that some of the points which a 40% sampling ratio mask did not sample were captured by other masks. In our experiment, different sampling masks provided their own information from their sampled points, meaning that four reconstruction tasks helped each other to achieve an efficient performance. Therefore, this explains why meta-learning is still superior to conventional learning when the sampling ratio is large.

Meta-learning expands a new paradigm for supervised learning—the purpose is to quickly learn multiple tasks. Meta-learning only learns task-invariant parameters once for a common feature that can be shared with four different tasks, and each $\sigma (\omega_{i})$ provides task-specific weighting parameters according to the principle of “learning to learn”. In conventional learning, the network parameter needs to be trained four times with four different masks since the task-invariant parameter cannot be generalized to other tasks, which is time-intensive. From Table 1 and Table 2, we observe that a small CS ratio needs a higher value of $\sigma (\omega_{i})$. In fact, in our model (11), the task-specific parameters behave as weighted constraints for task-specific regularizers, and the tables indicate that lower CS ratios require larger weights to be applied for the regularization.

A qualitative comparison between conventional and meta-learning methods is shown in Figure 1 and Figure 2, displaying the reconstructed MR images of the same slice for T1 and T2, respectively. We label the zoomed-in details of HGG in the red boxes. We observe evidence that conventional learning is more blurry and loses sharp edges, especially with lower CS ratios. From the point-wise error map, we find that meta-learning has the ability to reduce noises, especially in some detailed and complicated regions, compared to conventional learning.

We also tested the performance of meta-learning with two-thirds of the training and validation data for the T1-weighted image used in the previous experiment, denoted as “meta-learning”. For conventional learning, the network was also trained by using two-thirds of the training samples in the previous experiment. The testing dataset remained the same as before. These results are displayed in Table 1, where we denote the reduced data experiments as “meta-learning${}^{*}$” and “conventional${}^{*}$”. These experiments reveal that the accuracy of test data decreases when we reduce the training data size, but it is not a surprise that meta-learnining${}^{*}$ still outperforms conventional learning${}^{*}$, and even conventional learning.

To verify the reconstruction performance of the proposed LOA 1, we compared the proposed conventional learning with ISTA-Net${}^{+}$[54], which is a state-of-the-art deep unfolded network for MRI reconstruction. We retrained ISTA-Net${}^{+}$ with the same training dataset and testing dataset as conventional learning on the T1-weighted image. For a fair comparison, we used the same number of convolution kernels, the same dimension of kernels for each convolution during training, and the same phase numbers as conventional learning. The testing numerical results are listed in Table 1 and the MRI reconstructions are displayed in Figure 1. We can observe that the conventional learning which unrolls Algorithm 1 outperforms ISTA-Net${}^{+}$ in any of the CS ratios. From the corresponding point-wise absolute error, the conventional learning attains a much lower error and much better reconstruction quality.

### 5.4. Experimental Results with Different Unseen CS Ratios in Different Sampling Patterns

In this section, we test the generalizability of the proposed model for unseen tasks. We fixed the well-trained task-invariant parameter θ and only trained $\omega_{i}$ for sampling ratios of 15%, 25%, and 35% with radial masks and sampling ratios of 10%, 20%, 30%, and 40% with Cartesian masks. In this experiment, we only used 100 training data points for each CS ratio and applied a total of 50 epochs. The averaged evaluation values and standard deviations are listed in Table 3 and Table 4 for reconstructed T1 and T2 brain images, respectively, with radial masks, and Table 5 shows the qualitative performance for the reconstructed T2 brain image with random Cartesian sampling masks applied. In the T1 image reconstruction results, meta-learning showed an improvement of 1.6921 dB in PSNR for the 15% CS ratio, 1.6608 dB for the 25% CS ratio, and 0.5764 dB for the 35% ratio compared to the conventional method, showing the tendency that the level of reconstruction quality for lower CS ratios improved more than higher CS ratios. A similar trend was found for T2 reconstruction results with different sampling masks. The qualitative comparisons are illustrated in Figure 3, Figure 4 and Figure 5 for T1 and T2 images tested with unseen CS ratios in radial masks and T2 images tested with Cartesian masks with regular CS ratios, respectively. In the experiments conducted with radial masks, meta-learning was superior to conventional learning, especially at a CS ratio of 15%—one can observe that the detailed regions in red boxes maintained their edges and were closer to the true image, while the conventional method reconstructions are hazier and lost details in some complicated tissues. The point-wise error map also indicates that meta-learning has the ability to suppress noises.

Training with Cartesian masks is more difficult than radial masks, especially for conventional learning, where the network is not very deep since the network only applies three convolutions each with four kernels. Table 5 indicates that the average performance of Meta-learning improved about 1.87 dB compared to conventional methods with T2 brain images. These results further demonstrate that meta-learning has the benefit of parameter efficiency, and the performance is much better than conventional learning even if we apply a shallow network with a small amount of training data.

The numerical experimental results discussed above show that meta-learning is capable of fast adaption to new tasks and has more robust generalizability for a broad range of tasks with heterogeneous, diverse data. Meta-learning can be considered as an efficient technique for solving difficult tasks by leveraging the features extracted from easier tasks.

**Figure 6:**
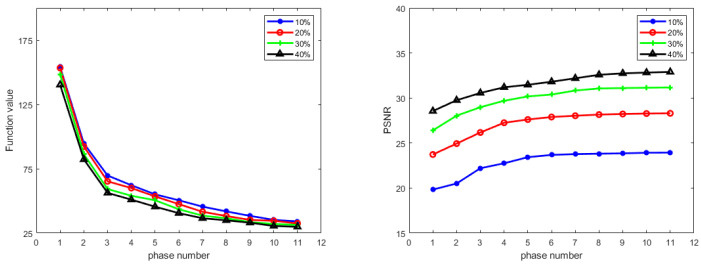
Convergence behavior of Algorithm 1 on T1 weighted MRI image reconstruction with four different CS ratios using radial mask. **Left**: Objective function value ϕ versus phase number. **Right**: PSNR value versus phase number.

Next, we empirically demonstrate the convergence of Algorithm 1 in Figure 6. This shows that the objective function value ϕ decreases and the PSNR value for testing data increases steadily as the number of phases increases, which indicates that the learned algorithm is indeed minimizing the learned function as we desired.

### 5.5. Future Work and Open Challenges

Deep optimization-based meta-learning techniques have shown great generalizability, but there are several open challenges that can be discussed and can potentially be addressed in future work. A major issue is the memorization problem, since the base learner needs to be optimized for a large number of phases and the training algorithm contains multiple gradient steps; furthermore, the computation is very expensive in terms of time and memory costs. In addition to reconstructing MRI through different trajectories, another potential application for medical imaging could be multi-modality reconstruction and synthesis. Capturing images of anatomy with multi-modality acquisitions enhances the diagnostic information and could be cast as a multi-task problem that could benefit from meta-learning.

## 6. Conclusions

In this paper, we put forward a novel deep model for MRI reconstructions via meta-learning. The proposed method has the ability to solve multi-tasks synergistically, and the well-trained model could generalize well to new tasks. Our baseline network is constructed by unfolding an LOA, which inherits the convergence property, improves the interpretability, and promotes the parameter efficiency of the designed network structure. The designated adaptive regularizer consists of a task-invariant learner and task-specific meta-knowledge. Network training follows a bilevel optimization algorithm that minimizes task-specific parameter ω in the upper level for the validation data and minimizes task-invariant parameters θ in the lower level for the training data with fixed ω. The proposed approach is the first model designed to solve the inverse problem by applying meta-training on the adaptive regularization in the variational model. We consider the recovery of undersampled raw data across different sampling trajectories with various sampling patterns as different tasks. Extensive numerical experiments on various MRI datasets demonstrate that the proposed method generalizes well at various sampling trajectories and is capable of fast adaption to unseen trajectories and sampling patterns. The reconstructed images achieve higher quality compared to conventional supervised learning for both seen and unseen k-space trajectory cases.

## Figures and Tables

**Table 1:** Quantitative evaluations of the reconstructions of T1 brain image associated with various sampling ratios of **radial** masks. Conventional${}^{*}$ and meta-learning${}^{*}$ are trained with two-thirds of the dataset used in training conventional and meta-learning approaches, respectively.

CS Ratio	Methods	PSNR	SSIM	NMSE	$\sigma (\omega_{i})$
	ISTA-Net${}^{+}$ [54]	21.2633 ± 1.0317	0.5487 ± 0.0440	0.1676 ± 0.0253	
	Conventional${}^{*}$	21.6947 ± 1.0264	0.5689 ± 0.0404	0.1595 ± 0.0240	
10%	Conventional	21.7570 ± 1.0677	0.5650 ± 0.0412	0.0259 ± 0.0082	
	Meta-learning${}^{*}$	22.9633 ± 1.0969	0.5962 ± 0.0415	0.0194 ± 0.0065	0.9339
	Meta-learning	23.2672 ± 1.1229	0.6101 ± 0.0436	0.0184 ± 0.0067	0.9218
	ISTA-Net${}^{+}$ [54]	26.2734 ± 1.0115	0.7068 ± 0.0364	0.0944 ± 0.0155	
	Conventional${}^{*}$	26.4639 ± 1.0233	0.7107 ± 0.0357	0.0924 ± 0.0154	
20%	Conventional	26.6202 ± 1.1662	0.7121 ± 0.0397	0.0910 ± 0.0169	
	Meta-learning${}^{*}$	27.9381 ± 1.1121	0.7541 ± 0.0360	0.0063 ± 0.0023	0.8150
	Meta-learning	28.2944 ± 1.2119	0.7640 ± 0.0377	0.0058 ± 0.0022	0.7756
	ISTA-Net${}^{+}$ [54]	28.8309 ± 1.3137	0.7492 ± 0.0407	0.0708 ± 0.0142	
	Conventional${}^{*}$	29.2923 ± 1.3194	0.7522 ± 0.0399	0.0671 ± 0.0136	
30%	Conventional	29.5034 ± 1.4446	0.7557 ± 0.0408	0.0657 ± 0.0143	
	Meta-learning${}^{*}$	30.8691 ± 1.5897	0.8310 ± 0.0394	0.0033 ± 0.0015	0.6359
	Meta-learning	31.1417 ± 1.5866	0.8363 ± 0.0385	0.0031 ± 0.0014	0.6501
	ISTA-Net${}^{+}$ [54]	30.7282 ± 1.5482	0.8008 ± 0.0428	0.0572 ± 0.0127	
	Conventional${}^{*}$	31.3761 ± 1.5892	0.8035 ± 0.0420	0.0532 ± 0.0121	
40%	Conventional	31.4672 ± 1.6390	0.8111 ± 0.0422	0.0029 ± 0.0014	
	Meta-learning${}^{*}$	32.7330 ± 1.6386	0.8623 ± 0.0358	0.0022 ± 0.0010	0.6639
	Meta-learning	32.8238 ± 1.7039	0.8659 ± 0.0370	0.0022 ± 0.0010	0.6447

**Table 2:** Quantitative evaluations of the reconstructions of T2 brain image associated with various sampling ratios of **radial** masks.

CS Ratio	Methods	PSNR	SSIM	NMSE	$\sigma (\omega_{i})$
10%	Conventional	23.0706 ± 1.2469	0.5963 ± 0.0349	0.2158 ± 0.0347	
	Meta-learning	24.0842 ± 1.3863	0.6187 ± 0.0380	0.0112 ± 0.0117	0.9013
20%	Conventional	27.0437 ± 1.0613	0.6867 ± 0.0261	0.1364 ± 0.0213	
	Meta-learning	28.9118 ± 1.0717	0.7843 ± 0.0240	0.0122 ± 0.0030	0.8742
30%	Conventional	29.5533 ± 1.0927	0.7565 ± 0.0265	0.1023 ± 0.0166	
	Meta-learning	31.4096 ± 0.9814	0.8488 ± 0.0217	0.0069 ± 0.0019	0.8029
40%	Conventional	32.0153 ± 0.9402	0.8139 ± 0.0238	0.0770 ± 0.0128	
	Meta-learning	33.1114 ± 1.0189	0.8802 ± 0.0210	0.0047 ± 0.0015	0.7151

**Table 3:** Quantitative evaluations of the reconstructions of T1 brain image associated with various sampling ratios of **radial** masks. Meta-learning was trained with CS ratios of 10%, 20%, 30%, and 40% and tested with unseen ratios of 15%, 25%, and 35%. The conventional method was subjected to regular training and testing with the same CS ratios of 15%, 25%, and 35%.

CS Ratio	Methods	PSNR	SSIM	NMSE	$\sigma (\omega_{i})$
15%	Conventional	24.6573 ± 1.0244	0.6339 ± 0.0382	0.1136 ± 0.0186	
	Meta-learning	26.3494 ± 1.0102	0.7088 ± 0.0352	0.0090 ± 0.0030	0.9429
25%	Conventional	28.4156 ± 1.2361	0.7533 ± 0.0368	0.0741 ± 0.0141	
	Meta-learning	30.0764 ± 1.4645	0.8135 ± 0.0380	0.0040 ± 0.0017	0.8482
35%	Conventional	31.5320 ± 1.5242	0.7923 ± 0.0420	0.0521 ± 0.0119	
	Meta-learning	32.1084 ± 1.6481	0.8553 ± 0.0379	0.0025 ± 0.0011	0.6552

**Table 4:** Quantitative evaluations of the reconstructions of T2 brain image associated with various sampling ratios of **radial** masks. Meta-learning was trained with CS ratios of 10%, 20%, 30%, and 40% and tested with unseen ratios of 15%, 25%, and 35%. Conventional method was subjected to regular training and testing with the same CS ratios of 15%, 25%, and 35%.

CS Ratio	Methods	PSNR	SSIM	NMSE	$\sigma (\omega_{i})$
15%	Conventional	24.8921 ± 1.2356	0.6259 ± 0.0285	0.1749 ± 0.0280	
	Meta-learning	26.7031 ± 1.2553	0.7104 ± 0.0318	0.0205 ± 0.0052	0.9532
25%	Conventional	29.0545 ± 1.1980	0.7945 ± 0.0292	0.1083 ± 0.0173	
	Meta-learning	30.0698 ± 0.9969	0.8164 ± 0.0235	0.0093 ± 0.0022	0.8595
35%	Conventional	31.5201 ± 1.0021	0.7978 ± 0.0236	0.0815 ± 0.0129	
	Meta-learning	32.0683 ± 0.9204	0.8615 ± 0.0209	0.0059 ± 0.0014	0.7388

**Table 5:** Quantitative evaluations of the reconstructions of T2 brain image associated with various sampling ratios of random **Cartesian** masks.

CS Ratio	Methods	PSNR	SSIM	NMSE	$\sigma (\omega_{i})$
10%	Conventional	20.8867 ± 1.2999	0.5082 ± 0.0475	0.0796 ± 0.0242	
	Meta-learning	22.0434 ± 1.3555	0.6279 ± 0.0444	0.0611 ± 0.0188	0.9361
20%	Conventional	22.7954 ± 1.2819	0.6057 ± 0.0412	0.0513 ± 0.0157	
	Meta-learning	24.7162 ± 1.3919	0.6971 ± 0.0380	0.0329 ± 0.0101	0.8320
30%	Conventional	24.2170 ± 1.2396	0.6537 ± 0.0360	0.0371 ± 0.0117	
	Meta-learning	26.4537 ± 1.3471	0.7353 ± 0.0340	0.0221 ± 0.0068	0.6771
40%	Conventional	25.3668 ± 1.3279	0.6991 ± 0.0288	0.1657 ± 0.0265	
	Meta-learning	27.5367 ± 1.4107	0.7726 ± 0.0297	0.0171 ± 0.0050	0.6498

## Data Availability

The data presented in this study are available on Multimodal Brain Tumor Segmentation Challenge (BraTs) 2018 [63] at https://www.med.upenn.edu/sbia/brats2018/data.html (accessed on 29 October 2021).

## Appendix A. Convergence Analysis

We make the following assumptions regarding *f* and g throughout this work:

- (a1): *f* is differentiable and (possibly) nonconvex, and ∇f is $L_{f}$-Lipschitz continuous.
- (a2): Every component of g is differentiable and (possibly) nonconvex, and ∇g is $L_{g}$-Lipschitz continuous.
- (a3): $\sup_{x\in X}∥\nabla g\left(x\right)∥\leq M$ for some constant M>0.
- (a4): ϕ is coercive, and $\phi^{*}=\min_{x\in X}\phi \left(x\right)>-\infty$.

First, we state the Clark subdifferential [13] of r(x) in Lemma A1, and we show that the gradient of $r_{\varepsilon }$ is Lipschitz continuous in Lemma A2.

**Lemma** **A1.** *Let r(x) be defined in* (4)*; then, the Clarke subdifferential of r at x is *

(A1) $$\partial r\left(x\right)=\{\sum_{j\in I_{0}}\nabla g_{i}{\left(x\right)}^{⊤}w_{j}+\sum_{j\in I_{1}}\nabla g_{j}{\left(x\right)}^{⊤}\frac{g_{j}\left(x\right)}{∥g_{j}\left(x\right)∥}\;|\;w_{j}\in R^{d},\;∥\Pi \left(w_{j};C\left(\nabla g_{i}\left(x\right)\right)\right)∥\leq 1,\;\forall \;j\in I_{0}\},$$

*where $I_{0}=\{j\in \left[m\right]\;|\;∥g_{j}\left(x\right)∥=0\}$, $I_{1}=\left[m\right]\setminus I_{0}$, and Π(w;C(A)) is the projection of w onto C(A) which stands for the column space of A.*

**Lemma** **A2.**
*The gradient of $r_{\varepsilon }$ is Lipschitz continuous with constant $m(L_{g}+\frac{2M^{2}}{\varepsilon })$.*

**Proof.** From $r_{\varepsilon }\left(x\right)=\sum_{j=1}^{m}(∥g_{j}\left(x\right){{∥}^{2}+\varepsilon^{2})}^{\frac{1}{2}}-\varepsilon$, it follows that

(A2) $$\nabla r_{\varepsilon }\left(x\right)=\sum_{j=1}^{m}\nabla g_{j}{\left(x\right)}^{⊤}g_{j}\left(x\right)(∥g_{j}\left(x\right){{∥}^{2}+\varepsilon^{2})}^{-\frac{1}{2}}.$$

For any $x_{1},x_{2}\in X$, we first define $h\left(x\right)=g_{j}\left(x\right)(∥g_{j}\left(x\right){{∥}^{2}+\varepsilon^{2})}^{-\frac{1}{2}}$, so $∥h(x)∥<1$.

(A3a) $$\begin{array}{cc} \; & ∥h\left(x_{1}\right)-h\left(x_{2}\right)∥ \end{array}$$

(A3b) $$\begin{array}{cc} \; & =∥\frac{g_{j}\left(x_{1}\right)}{\sqrt{∥g_{j}\left(x_{1}\right){∥}^{2}+\varepsilon^{2}}}-\frac{g_{j}\left(x_{2}\right)}{\sqrt{∥g_{j}\left(x_{2}\right){∥}^{2}+\varepsilon^{2}}}∥ \end{array}$$

(A3c) $$\begin{array}{cc} \; & =∥\frac{g_{j}\left(x_{1}\right)}{\sqrt{∥g_{j}\left(x_{1}\right){∥}^{2}+\varepsilon^{2}}}-\frac{g_{j}\left(x_{1}\right)}{\sqrt{∥g_{j}\left(x_{2}\right){∥}^{2}+\varepsilon^{2}}}+\frac{g_{j}\left(x_{1}\right)}{\sqrt{∥g_{j}\left(x_{2}\right){∥}^{2}+\varepsilon^{2}}}-\frac{g_{j}\left(x_{2}\right)}{\sqrt{∥g_{j}\left(x_{2}\right){∥}^{2}+\varepsilon^{2}}}∥ \end{array}$$

(A3d) $$\begin{array}{cc} \; & \leq ∥g_{j}\left(x_{1}\right)\left(\frac{\sqrt{∥g_{j}\left(x_{2}\right){∥}^{2}+\varepsilon^{2}}-\sqrt{∥g_{j}\left(x_{1}\right){∥}^{2}+\varepsilon^{2}}}{\sqrt{∥g_{j}\left(x_{1}\right){∥}^{2}+\varepsilon^{2}}\sqrt{∥g_{j}\left(x_{2}\right){∥}^{2}+\varepsilon^{2}}}\right)∥+∥\frac{g_{j}\left(x_{1}\right)-g_{j}\left(x_{2}\right)}{\sqrt{∥g_{j}\left(x_{2}\right){∥}^{2}+\varepsilon^{2}}}∥ \end{array}$$

(A3e) $$\begin{array}{cc} \; & \leq ∥\frac{g_{j}\left(x_{1}\right)}{\sqrt{∥g_{j}\left(x_{1}\right){∥}^{2}+\varepsilon^{2}}}∥∥\frac{\sqrt{∥g_{j}\left(x_{2}\right){∥}^{2}+\varepsilon^{2}}-\sqrt{∥g_{j}\left(x_{1}\right){∥}^{2}+\varepsilon^{2}}}{\sqrt{∥g_{j}\left(x_{2}\right){∥}^{2}+\varepsilon^{2}}}∥+\frac{1}{\varepsilon }∥g_{j}\left(x_{1}\right)-g_{j}\left(x_{2}\right)∥ \end{array}$$

(A3f) $$\begin{array}{cc} \; & \leq \frac{1}{\varepsilon }∥\sqrt{∥g_{j}\left(x_{2}\right){∥}^{2}+\varepsilon^{2}}-\sqrt{∥g_{j}\left(x_{1}\right){∥}^{2}+\varepsilon^{2}}∥+\frac{1}{\varepsilon }∥g_{j}\left(x_{1}\right)-g_{j}\left(x_{2}\right)∥ \end{array}$$

(A3g) $$\begin{array}{cc} \; & \leq \frac{1}{\varepsilon }\frac{∥g_{j}\left(x_{2}\right){∥}^{2}-{∥g_{j}\left(x_{1}\right)∥}^{2}}{\sqrt{∥g_{j}\left(x_{2}\right){∥}^{2}+\varepsilon^{2}}+\sqrt{∥g_{j}\left(x_{1}\right){∥}^{2}+\varepsilon^{2}}}+\frac{1}{\varepsilon }∥g_{j}\left(x_{1}\right)-g_{j}\left(x_{2}\right)∥ \end{array}$$

(A3h) $$\begin{array}{cc} \; & \leq \frac{1}{\varepsilon }\underset{<1}{\underbrace{\frac{∥g_{j}\left(x_{2}\right)∥+∥g_{j}\left(x_{1}\right)∥}{\sqrt{∥g_{j}\left(x_{2}\right){∥}^{2}+\varepsilon^{2}}+\sqrt{∥g_{j}\left(x_{1}\right){∥}^{2}+\varepsilon^{2}}}}}\left(∥g_{j}\left(x_{2}\right)∥-∥g_{j}\left(x_{1}\right)∥\right)+\frac{1}{\varepsilon }∥g_{j}\left(x_{1}\right)-g_{j}\left(x_{2}\right)∥ \end{array}$$

(A3i) $$\begin{array}{cc} \; & \leq \frac{1}{\varepsilon }∥g_{j}\left(x_{2}\right)-g_{j}\left(x_{1}\right)∥+\frac{1}{\varepsilon }∥g_{j}\left(x_{1}\right)-g_{j}\left(x_{2}\right)∥ \end{array}$$

(A3j) $$\begin{array}{cc} \; & =\frac{2}{\varepsilon }∥g_{j}\left(x_{1}\right)-g_{j}\left(x_{2}\right)∥. \end{array}$$

where to obtain (A3f) we used $∥\frac{g_{j}\left(x_{1}\right)}{\sqrt{∥g_{j}\left(x_{1}\right){∥}^{2}+\varepsilon^{2}}}∥<1\;\mathrm{and}\;\frac{1}{\sqrt{∥g_{j}\left(x_{1}\right){∥}^{2}+\varepsilon^{2}}}<\frac{1}{\varepsilon }$. Therefore, we have

(A4a) $$\begin{array}{cc} \; & ∥\nabla r_{\varepsilon }\left(x_{1}\right)-\nabla r_{\varepsilon }\left(x_{2}\right)∥ \end{array}$$

(A4b) $$\begin{array}{cc} \; & =\sum_{j=1}^{m}∥\nabla g_{j}{\left(x_{1}\right)}^{⊤}h\left(x_{1}\right)-\nabla g_{j}{\left(x_{2}\right)}^{⊤}h\left(x_{2}\right)∥ \end{array}$$

(A4c) $$\begin{array}{cc} \; & =\sum_{j=1}^{m}∥\nabla g_{j}{\left(x_{1}\right)}^{⊤}h\left(x_{1}\right)-\nabla g_{j}{\left(x_{2}\right)}^{⊤}h\left(x_{1}\right)+\nabla g_{j}{\left(x_{2}\right)}^{⊤}h\left(x_{1}\right)-\nabla g_{j}{\left(x_{2}\right)}^{⊤}h\left(x_{2}\right)∥ \end{array}$$

(A4d) $$\begin{array}{cc} \; & \leq \sum_{j=1}^{m}∥{\left(\nabla g_{j}\left(x_{1}\right)-\nabla g_{j}\left(x_{2}\right)\right)}^{⊤}h\left(x_{1}\right)∥+∥\nabla g_{j}\left(x_{2}\right)\left(h\left(x_{1}\right)-h\left(x_{2}\right)\right)∥\\ \; & \leq \sum_{j=1}^{m}∥\nabla g_{j}\left(x_{1}\right)-\nabla g_{j}\left(x_{2}\right)∥∥h(x_{1})∥+\nabla g_{j}\left(x_{2}\right)∥h\left(x_{1}\right)-h\left(x_{2}\right)∥ \end{array}$$

(A4e) $$\begin{array}{cc} \; & \leq \sum_{j=1}^{m}∥\nabla g_{j}\left(x_{1}\right)-\nabla g_{j}\left(x_{2}\right)∥+∥\nabla g_{j}\left(x_{2}\right)∥\frac{2}{\varepsilon }∥g_{j}\left(x_{1}\right)-g_{j}\left(x_{2}\right)∥\;\mathrm{by}\;\left(A3\right)\;\mathrm{and}\; \end{array}$$

(A4f) $$\begin{array}{cc} \; & \leq m(L_{g}∥x_{1}-x_{2}∥+M\frac{2}{\varepsilon }\cdot M∥x_{1}-x_{2}∥), \end{array}$$

where the first term of the last inequality is due to the $L_{g}$-Lipschitz continuity of $\nabla g_{j}$. The second term is because of $∥g_{j}\left(x_{1}\right)-g_{j}\left(x_{2}\right)∥=∥\nabla g_{j}\left(\tilde{x}\right)\left(x_{1}-x_{2}\right)∥$ for some $\tilde{x}\in X$ due to the mean value theorem and $∥\nabla g_{j}\left(\tilde{x}\right)∥\leq \sup_{x\in X}∥\nabla g_{j}\left(x\right)∥\leq M$. Therefore, we obtain

(A5) $$∥\nabla r_{\varepsilon }\left(x_{1}\right)-\nabla r_{\varepsilon }\left(x_{2}\right)∥\leq m\left(L_{g}+\frac{2M^{2}}{\varepsilon }\right)∥x_{1}-x_{2}∥.$$

□

**Lemma** **A3.**
*Let $\varepsilon ,\eta ,\tau_{t},a>0$, 0<ρ<1, and choose the initial $x_{0}\in X$. Suppose the sequence $\left\{x_{t}\right\}$ is generated by executing Lines 3–14 of Algorithm 1 with fixed $\varepsilon_{t}=\varepsilon$ and that $0<\delta <\frac{L_{\varepsilon }}{2/a+L_{\varepsilon }}<1$ exists such that $\alpha_{t}\geq \frac{\delta }{L_{\varepsilon }}>0$, where $L_{\varepsilon }=L_{f}+L_{h}+mL_{g}+\frac{2M^{2}}{\varepsilon }$ and $\phi^{*}:=\min_{x\in X}\phi \left(x\right)$. Then, the following statements hold:*

1. *$∥\nabla \phi_{\varepsilon }\left(x_{t}\right)∥\to 0$ as t→∞.*
2. *$\max\left\{t\in N\;|\;\nabla \phi_{\varepsilon }\left(x_{t+1}\right)\geq \eta \right\}\leq \max\left\{\frac{aL_{\varepsilon }^{2}}{\delta^{2}},a^{3}\right\}\{\phi_{\varepsilon }\left(x_{0}\right)-\phi^{*}+\varepsilon \}$.*

**Proof.** 1. In each iteration, we compute $u_{t+1}=z_{t+1}-\tau_{t}\sigma \left(\omega_{i}\right)\nabla r_{\varepsilon_{t}}\left(z_{t+1}\right)$.

1.1. In the case the condition
  (A6) $$∥\nabla \phi_{\varepsilon }\left(x_{t}\right)∥\leq a∥u_{t+1}-x_{t}∥\;\;\;and\;\;\;\phi_{\varepsilon }\left(u_{t+1}\right)-\phi_{\varepsilon }\left(x_{t}\right)\leq -\frac{1}{a}{∥u_{t+1}-x_{t}∥}^{2}$$
  holds with a>0, we put $x_{t+1}=u_{t+1}$, and we have $\phi_{\varepsilon }\left(u_{t+1}\right)\leq \phi_{\varepsilon }\left(x_{t}\right)$.
1.2. Otherwise, we compute $v_{t+1}=x_{t}-\alpha_{t}\nabla \phi_{\varepsilon }\left(x_{t}\right)$, where $\alpha_{t}$ is found through the line search until the criteria
  (A7) $$\phi_{\varepsilon }\left(v_{t+1}\right)-\phi_{\varepsilon }\left(x_{t}\right)\leq -\frac{1}{a}{∥v_{t+1}-x_{t}∥}^{2}$$
  holds, and then put $x_{t+1}=v_{t+1}$. From Lemma A2, we know that the gradient $\nabla r_{\varepsilon }\left(x\right)$ is Lipschitz continuous with constant $m(L_{g}+\frac{2M^{2}}{\varepsilon })$. Furthermore, we assumed in (a1) that ∇f is $L_{f}$-Lipschitz continuous. Hence, putting $L_{\varepsilon }=L_{f}+m\left(L_{g}+\frac{2M^{2}}{\varepsilon }\right)$, we find that $\nabla \phi_{\varepsilon }$ is $L_{\varepsilon }$-Lipschitz continuous, which implies
  (A8) $$\phi_{\varepsilon }\left(v_{t+1}\right)\leq \phi_{\varepsilon }\left(x_{t}\right)+\langle \nabla \phi_{\varepsilon }\left(x_{t}\right),v_{t+1}-x_{t}\rangle +\frac{L_{\varepsilon }}{2}{∥v_{t+1}-x_{t}∥}^{2}.$$
  Furthermore, by the optimality condition of
  $$v_{t+1}=\underset{x}{arg min}\langle \nabla f\left(x_{t}\right),x-x_{t}\rangle +\sigma \left(\omega_{i}\right)\langle \nabla r_{\varepsilon }\left(x_{t}\right),x-x_{t}\rangle +\frac{1}{2\alpha_{t}}{∥x-x_{t}∥}^{2},$$
  we have
  (A9) $$\langle \nabla \phi_{\varepsilon }\left(x_{t}\right),v_{t+1}-x_{t}\rangle +\frac{1}{2\alpha_{t}}{∥v_{t+1}-x_{t}∥}^{2}\leq 0.$$
  Combining (A8) and (A9) and $v_{t+1}=x_{t}-\alpha_{t}\nabla \phi_{\varepsilon }\left(x_{t}\right)$ in line 8 of Algorithm 1 yields
  (A10) $$\phi_{\varepsilon }\left(v_{t+1}\right)-\phi_{\varepsilon }\left(x_{t}\right)\leq -\left(\frac{1}{2\alpha_{t}}-\frac{L_{\varepsilon }}{2}\right){∥v_{t+1}-x_{t}∥}^{2}.$$
  Therefore, it is sufficient for $\alpha_{t}\leq \frac{1}{2/a+L_{\varepsilon }}$ for the criteria (A7) to be satisfied. This process only take finitely many iterations since we can find a finite *t* such that $\rho^{t}\alpha_{t}\leq \frac{1}{2/a+L_{\varepsilon }}$, and through the line search, we can obtain $\phi_{\varepsilon }\left(v_{t+1}\right)\leq \phi_{\varepsilon }\left(x_{t}\right)$.
  Therefore, in either case of 1.1. or 1.2. where we take $x_{t+1}=u_{t+1}$ or $v_{t+1}$, we can obtain
  (A11) $$\phi_{\varepsilon }\left(x_{t+1}\right)\leq \phi_{\varepsilon }\left(x_{t}\right),\;\mathrm{for}\;\mathrm{all}\;t\geq 0.$$
  Now, from case 1.1., (A6) gives
  (A12a) $$\begin{array}{cc} {∥\nabla \phi_{\varepsilon }\left(x_{t}\right)∥}^{2}\leq a^{2}{∥u_{t+1}-x_{t}∥}^{2}\leq & a^{3}\left(\phi_{\varepsilon }\left(x_{t}\right)-\phi_{\varepsilon }\left(u_{t+1}\right)\right), \end{array}$$
  (A12b) $$\begin{array}{cc} \mathrm{therefore}\;\mathrm{if}\;x_{t+1}=u_{t+1}\;\mathrm{we}\;\mathrm{get}\; & {\nabla \phi_{\varepsilon }\left(x_{t}\right)}^{2}\leq a^{3}\left(\phi_{\varepsilon }\left(x_{t}\right)-\phi_{\varepsilon }\left(x_{t+1}\right)\right). \end{array}$$
  From case 1.2. and $v_{t+1}=x_{t}-\alpha_{t}\nabla \phi_{\varepsilon }\left(x_{t}\right)$, we have
  (A13a) $$\begin{array}{cc} \phi_{\varepsilon }\left(v_{t+1}\right)-\phi_{\varepsilon }\left(x_{t}\right)\leq & -\frac{1}{a}{∥v_{t+1}-x_{t}∥}^{2}=-\frac{1}{a}\alpha_{t}^{2}{∥\nabla \phi_{\varepsilon }\left(x_{t}\right)∥}^{2} \end{array}$$
  (A13b) $$\begin{array}{cc} ⟹\; & {∥\nabla \phi_{\varepsilon }\left(x_{t}\right)∥}^{2}\leq \frac{a}{\alpha_{t}^{2}}\left(\phi_{\varepsilon }\left(x_{t}\right)-\phi_{\varepsilon }\left(v_{t+1}\right)\right), \end{array}$$
  (A13c) $$\begin{array}{cc} \;\mathrm{then}\;\mathrm{if}\;x_{t+1}=v_{t+1},\;\mathrm{we}\;\mathrm{have}\;\; & {∥\nabla \phi_{\varepsilon }\left(x_{t}\right)∥}^{2}\leq \frac{a}{\alpha_{t}^{2}}\left(\phi_{\varepsilon }\left(x_{t}\right)-\phi_{\varepsilon }\left(x_{t+1}\right)\right). \end{array}$$
  Since $\frac{\delta }{L_{\varepsilon }}\leq \alpha_{t}\leq \frac{1}{2/a+L_{\varepsilon }}$, we have
  (A14) $${∥\nabla \phi_{\varepsilon }\left(v_{t+1}\right)∥}^{2}\leq \frac{aL_{\varepsilon }^{2}}{\delta^{2}}\left(\phi_{\varepsilon }\left(v_{t+1}\right)-\phi_{\varepsilon }\left(x_{t+1}\right)\right).$$
  Combining (A12b) and (A14) and selecting $C=\max\{\frac{aL_{\varepsilon }^{2}}{\delta^{2}},a^{3}\}$, we obtain
  (A15) $${∥\nabla \phi_{\varepsilon }\left(x_{t}\right)∥}^{2}\leq C\left(\phi_{\varepsilon }\left(x_{t}\right)-\phi_{\varepsilon }\left(x_{t+1}\right)\right).$$
  Summing up (A15) for t=0,⋯,T, we have
  (A16) $$\sum_{t=0}^{T}{∥\nabla \phi_{\varepsilon }\left(x_{t}\right)∥}^{2}\leq C\left(\phi_{\varepsilon }\left(x_{0}\right)-\phi_{\varepsilon }\left(x_{T+1}\right)\right).$$
  Combined with the fact that $\phi_{\varepsilon }\left(x\right)\geq \phi \left(x\right)-\varepsilon \geq \phi^{*}-\varepsilon$ for every x∈X, we have
  (A17) $$\sum_{t=0}^{T}{∥\nabla \phi_{\varepsilon }\left(x_{t}\right)∥}^{2}\leq C\left(\phi_{\varepsilon }\left(x_{0}\right)-\phi^{*}+\varepsilon \right).$$
  The right-hand side is a finite constant, and hence by letting t→∞, we know that $∥\nabla \phi_{\varepsilon }\left(x_{t}\right)∥\to 0$, which proves the first statement.

2. Denote $\kappa :=\max\{t\in N\;|\;\nabla \phi_{\varepsilon }\left(x_{t+1}\right)\geq \eta \}$; then, we know that $\nabla \phi_{\varepsilon }\left(x_{t+1}\right)\geq \eta$ for all t≤κ−1. Hence, we have

(A18) $$\kappa \eta^{2}\leq \sum_{t=0}^{\kappa -1}{∥\nabla \phi_{\varepsilon }\left(x_{t+1}\right)∥}^{2}=\sum_{t=1}^{\kappa }{∥\nabla \phi_{\varepsilon }\left(x_{t}\right)∥}^{2}\leq C\left(\phi_{\varepsilon }\left(x_{0}\right)-\phi^{*}+\varepsilon \right).$$

which implies the second statement. □

**Lemma** **A4.**
*Suppose that the sequence $\left\{x_{t}\right\}$ is generated by Algorithm 1 with an initial guess $x_{0}$. Then, for any t≥0, we have $\phi_{\varepsilon_{t+1}}\left(x_{t+1}\right)+\varepsilon_{t+1}\leq \phi_{\varepsilon_{t}}\left(x_{t}\right)+\varepsilon_{t}$.*

**Proof.** To prove this statement, we can prove

(A19) $$\phi_{\varepsilon_{t+1}}\left(x_{t+1}\right)+\varepsilon_{t+1}\leq \phi_{\varepsilon_{t}}\left(x_{t+1}\right)+\varepsilon_{t}\leq \phi_{\varepsilon_{t}}\left(x_{t}\right)+\varepsilon_{t}.$$

The second inequality is immediately obtained from (A11). Now, we prove the first inequality. For any ε>0, denote

(A20) $$r_{\varepsilon ,j}\left(x\right)=\sqrt{∥g_{j}{\left(x\right)∥}_{2}^{2}+\varepsilon^{2}}-\varepsilon .$$

Since $\phi_{\varepsilon }\left(x\right)=f\left(x\right)+\sigma \left(\omega_{i}\right)\sum_{j=1}^{m}r_{\varepsilon ,j}\left(x\right)$, it suffices to show that

(A21) $$r_{\varepsilon_{t+1},j}\left(x_{t+1}\right)+\varepsilon_{t+1}\leq r_{\varepsilon_{t},j}\left(x_{t+1}\right)+\varepsilon_{t}$$

If $\varepsilon_{t+1}=\varepsilon_{t}$, then the two quantities above are identical and the first inequality holds. Now, suppose $\varepsilon_{t+1}=\gamma \varepsilon_{t}\leq \varepsilon_{t}$; then,

(A22) $$r_{\varepsilon_{t+1},j}\left(x_{t+1}\right)+\varepsilon_{t+1}=\sqrt{∥g_{j}{\left(x\right)∥}_{2}^{2}+\varepsilon_{t+1}^{2}}\leq \sqrt{∥g_{j}{\left(x\right)∥}_{2}^{2}+\varepsilon_{t}^{2}}=r_{\varepsilon_{t},j}\left(x_{t+1}\right)+\varepsilon_{t},$$

which implies the first inequality of (A19). □

**Theorem** **A5.**
*Suppose that $\left\{x_{t}\right\}$ is the sequence generated by Algorithm 1 with any initial $x_{0}$, $\epsilon_{\mathrm{tol}}=0$ and T=∞. Let $\left\{x_{t_{l}+1}\right\}$ be the subsequence that satisfies the reduction criterion in step 15 of Algorithm 1, i.e., $∥\nabla \phi_{\varepsilon_{t_{l}}}\left(x_{t_{l}+1}\right)∥\leq \sigma \varepsilon_{t_{l}}\gamma$ for $t=t_{l}$ and l=1,2,⋯. Then $\left\{x_{t_{l}+1}\right\}$ has at least one accumulation point, and every accumulation point of $\left\{x_{t_{l}+1}\right\}$ is a clarke stationary point of $\min_{x}\phi \left(x\right):=f\left(x\right)+\sigma \left(\omega_{i}\right)r\left(x\right)$.*

**Proof.** By Lemma A4 and $\phi \left(x\right)\leq \phi_{\varepsilon }\left(x\right)+\varepsilon$ for all ε>0 and x∈X, we know that

(A23) $$\phi \left(x_{t}\right)\leq \phi_{\varepsilon_{t}}\left(x_{t}\right)+\varepsilon_{t}\leq \cdots \leq \phi_{\varepsilon_{0}}\left(x_{0}\right)+\varepsilon_{0}<\infty .$$

Since ϕ is coercive, we know that $\left\{x_{t}\right\}$ is bounded, and the selected subsequence $\left\{x_{t_{l}+1}\right\}$ is also bounded and has at least one accumulation point. Note that $∥\nabla \phi_{\varepsilon_{t_{l}}}\left(x_{t_{l}+1}\right)∥\leq \sigma \varepsilon_{t_{l}}\gamma =\sigma \varepsilon_{0}\gamma^{l+1}\to 0$ as l→∞. Let $\left\{x_{p+1}\right\}$ be any convergent subsequence of $\left\{x_{t_{l}+1}\right\}$ and denote $\varepsilon_{p}$ as the corresponding $\varepsilon_{t}$ used in Algorithm 1 that generates $x_{p+1}$. Then, there exists $x^{*}\in X$ such that $x_{p+1}\to x^{*}$ as $\varepsilon_{p}\to 0,$ and $\nabla \phi_{\varepsilon_{p}}\left(x_{p+1}\right)\to 0$ as p→∞. Note that the Clarke subdifferential of ϕ at $x^{*}$ is given by $\partial \phi \left(x^{*}\right)=\partial f\left(x^{*}\right)+\sigma \left(\omega_{i}\right)\partial r\left(x^{*}\right)$:

(A24) $$\begin{array}{c} \partial \phi \left(\hat{x}\right)=\{\nabla f\left(\hat{x}\right)+\sigma \left(\omega_{i}\right)\sum_{j\in I_{0}}\nabla g_{j}{\left(\hat{x}\right)}^{⊤}w_{j}+\sigma \left(\omega_{i}\right)\sum_{j\in I_{1}}\nabla g_{j}{\left(\hat{x}\right)}^{⊤}\frac{g_{j}\left(\hat{x}\right)}{∥g_{j}\left(\hat{x}\right)∥}\;|\\ \;∥\Pi (w_{j};C\left(\nabla g_{j}\left(\hat{x}\right)\right))∥\leq 1,\;\forall \;j\in I_{0}\}, \end{array}$$

where $I_{0}=\{j\in \left[m\right]\;|\;∥g_{i}\left(\hat{x}\right)∥=0\}$ and $I_{1}=\left[m\right]\setminus I_{0}$. If $j\in I_{0}$, we have $∥g_{j}\left(x\right)∥=0\Leftrightarrow g_{j}\left(x\right)=0$: then,

(A25a) $$\begin{array}{cc} \partial r_{\varepsilon }\left(x\right) & =\sum_{j\in I_{0}}\nabla g_{j}{\left(x\right)}^{⊤}\frac{g_{j}\left(x\right)}{{\left({∥g_{j}\left(x\right)∥}^{2}+\varepsilon^{2}\right)}^{\frac{1}{2}}}+\sum_{j\in I_{1}}\nabla g_{j}{\left(x\right)}^{⊤}\frac{g_{j}\left(x\right)}{{\left({∥g_{j}\left(x\right)∥}^{2}+\varepsilon^{2}\right)}^{\frac{1}{2}}} \end{array}$$

(A25b) $$\begin{array}{cc} \; & =0+\sum_{j\in I_{1}}\nabla g_{j}{\left(x\right)}^{⊤}\frac{g_{j}\left(x\right)}{{\left({∥g_{j}\left(x\right)∥}^{2}+\varepsilon^{2}\right)}^{\frac{1}{2}}} \end{array}$$

Therefore, we obtain

(A26) $$\nabla \phi_{\varepsilon_{p}}\left(x_{p+1}\right)=\nabla f\left(x_{p+1}\right)+\sigma \left(\omega_{i}\right)\sum_{j\in I_{1}}\nabla g_{j}{\left(x\right)}^{⊤}\frac{g_{j}\left(x\right)}{{\left({∥g_{j}\left(x\right)∥}^{2}+\varepsilon_{p}^{2}\right)}^{\frac{1}{2}}}.$$

Comparing (A24) and (A26), we can see that the first term on the right-hand side of (A26) converges to that of (A24), due to the fact that $x_{p+1}\to \hat{x}$ and the continuity of ∇f. Together with the continuity of $g_{i}$ and $\nabla g_{i}$, the last term of (A24) converges to the last term of (A26) as $\varepsilon_{p}\to 0$ and $∥g_{j}\left(x\right)∥>0$. Furthermore, apparently 0 is a special case of the second term in (A26). Hence, we know that

$$\mathrm{dist}(\nabla \phi_{\varepsilon_{p}}\left(x_{p+1}\right),\partial \phi \left(\hat{x}\right))\to 0,$$

as p→∞. Since $\nabla \phi_{\varepsilon_{p}}\left(x_{p+1}\right)\to 0$ and $\partial \phi (\hat{x})$ is closed, we conclude that $0\in \partial \phi (\hat{x})$. □
